# Chchd10: A Novel Metabolic Sensor Modulating Adipose Tissue Homeostasis

**DOI:** 10.1002/advs.202408763

**Published:** 2025-02-22

**Authors:** Xiaoping Wu, Zixuan Zhang, Jingjing Li, Jiuyu Zong, Lufengzi Yuan, Lingling Shu, Lai Yee Cheong, Xiaowen Huang, Mengxue Jiang, Zhihui Ping, Aimin Xu, Ruby L.C. Hoo

**Affiliations:** ^1^ State Key Laboratory of Pharmaceutical Biotechnology The University of Hong Kong Hong Kong SAR China; ^2^ Department of Pharmacology and Pharmacy The University of Hong Kong Hong Kong SAR China; ^3^ Department of Rehabilitation Sciences Faculty of Health and Social Sciences Hong Kong Polytechnic University Hong Kong SAR China; ^4^ State Key Laboratory of Oncology in South China Guangdong Provincial Clinical Research Center for Cancer Department of Hematological Oncology Sun Yat‐sen University Cancer Center China; ^5^ Department of Medicine The University of Hong Kong Hong Kong SAR China

**Keywords:** adipogenesis, adipose tissue remodeling, Chchd10, GSTA4, NRF2, obesity, p62, protein carbonylation, TDP43

## Abstract

Dysregulation of adipose tissue (AT) homeostasis in obesity contributes to metabolic stress and disorders. Here, we identified that Coiled‐coil‐helix‐coiled‐coil‐helix domain containing 10 (Chchd10) is a novel regulator of AT remodeling upon excess energy intake. Chchd10 is significantly reduced in the white adipose tissue (WAT) of mice in response to high‐fat diet (HFD) feeding. AT‐Chchd10 deficiency accelerates adipogenesis predominantly in subcutaneous AT of mice to store excess energy in response to short‐term HFD feeding while upregulates glutathione S‐transferase A4 (GSTA4) to facilitate 4‐HNE clearance mainly in visceral AT to prevent protein carbonylation‐induced cell dysfunction after long‐term HFD feeding. Hence, Chchd10 deficiency attenuates diet‐induced obesity and related metabolic disorders in mice. Mechanistically, Chchd10 deficiency enhances adipogenesis and GSTA4 expression by activating TDP43/Raptor/p62/Keap1/NRF2 axis. Notably, the beneficial effect of Chchd10 deficiency is eliminated in hypertrophic adipocytes, where p62 is strikingly reduced. Collectively, Chchd10 is a metabolic sensor maintaining AT homeostasis, and the loss of p62 in adipose tissue under obese conditions impairs Chchd10‐mediated AT remodeling.

## Graphical abstract

Chchd10 is a novel regulator of adipose tissue (AT) homeostasis upon excess energy intake. Rapid Chchd10 reduction in response to high‐fat diet induction promotes adipogenesis in subcutaneous AT to store excess energy. On the other hand, sustained Chchd10 reduction protects visceral AT from 4‐HNE‐induced adipocyte dysfunction, attenuating the development of obesity and related metabolic disorders.

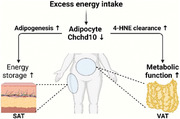

## 1 Introduction

Adipose tissue homeostasis is crucial for maintaining energy balance and metabolic health in the body. In response to excess energy intake, adipose tissue is remodeled by adipogenesis, which encompasses hyperplasia (the generation of new adipocytes) and hypertrophy (increasing the size of existing adipocytes), contributing to the expansion of adipose tissue to store energy as fat^[^ ^1^, ^2^ ^]^ thus maintaining homeostasis. Adipocyte hyperplasia predominantly occurs in subcutaneous adipose tissue (SAT), which consists of small adipocytes and is regarded as the largest and least detrimental site for storing excess calories. However, as hyperplasia reaches its limit over time, excessive fat accumulation in visceral adipose tissue (VAT), which relies on adipocyte hypertrophy.^[^ ^3^ ^]^ In humans, central obesity characterized by excessive VAT is closely associated with metabolic complications, including insulin resistance, dyslipidemia, and chronic low‐grade inflammation.^[^ ^4^, ^5^ ^]^ This is because hypertrophic adipocytes lose their functions and become less insulin sensitive with aberrant production of adipokines, increased lipolysis, and diminished fat‐storing capacity.^[^ ^2^ ^]^ Hypertrophic adipocytes also cause hypoxia, leading to the infiltration of pro‐inflammatory macrophages and fibrosis, which collectively contribute to the development of rigid dysfunctional adipose tissue.^[^ ^2^ ^]^ Generally, aberrant adipose tissue remodeling disrupts adipose tissue homeostasis, leading to obesity‐related metabolic disorders.

Redox imbalance, characterized by increased reactive oxygen species (ROS) or reduced antioxidant defenses, is an instigator disrupting adipose tissue homeostasis. Excessive ROS suppresses adipocyte differentiation, enhances hypertrophy, and alters adipocyte physiological functions.^[^ ^6^ ^]^ Protein carbonylation is a detrimental event, which is mainly mediated by 4‐Hydroxynonenal (4‐HNE), a reactive aldehyde generated from ROS‐induced lipid peroxidation.^[^ ^7^ ^]^ Carbonylation leads to protein dysfunction and aggregation^[^ ^8^, ^9^ ^]^ and is positively associated with increased adiposity^[^ ^10^ ^]^ and insulin resistance^[^ ^11^ ^]^ in patients with obesity. In adipocytes, increased carbonylation of mitochondrial proteins reduces oxidative capacity, leading to impaired mitochondrial function and enhanced ROS synthesis,^[^ ^12^ ^]^ which undermines vital metabolic processes, including adipokine production, lipogenesis, and fatty acid oxidation,^[^ ^13^ ^]^ and forms a detrimental loop. Carbonylation of nuclear zinc finger proteins also suppresses the transcription of genes responsible for mitochondrial bioenergetics, further impairing adipocyte functions.^[^ ^14^ ^]^ Moreover, carbonylation of insulin receptors and glycolytic enzymes impairs insulin sensitivity and glucose metabolism in adipocytes.^[^ ^15^ ^]^ In both obese humans and mice, a significant elevation of 4‐HNE level was observed in their VAT but not SAT,^[^ ^16^, ^17^ ^]^ suggesting that 4‐HNE‐induced protein carbonylation in VAT may serve as a causal link between central obesity and metabolic complications. On the other hand, reduced antioxidant defenses in adipose tissue of both humans and rodents are suggested to potentiate obesity and insulin resistance.^[^ ^17^, ^18^, ^19^ ^]^ In premenopausal obese women, decreased glutathione (GSH) metabolism, a crucial endogenous antioxidant maintaining redox balance by neutralizing ROS or clearing electrophilic compounds including 4‐HNE, was observed in both SAT and VAT.^[^ ^17^ ^]^ Nevertheless, the causal factors triggering the reduction of the natural antioxidant defenses remain unclear.

By analyzing the RNA sequencing dataset of white adipocytes from mice subjected to short‐term high‐fat diet (HFD) feeding and standard chow (STC)‐feeding,^[^ ^20^ ^]^ we identified Coiled‐coil‐helix‐coiled‐coil‐helix domain containing 10 (Chchd10), a nuclear‐encoded mitochondrial protein^[^ ^21^ ^]^ as one of the most downregulated genes upon HFD induction. Missense mutations of Chchd10 are implicated in neurodegenerative diseases.^[^ ^21^ ^]^ Mutant Chchd10 aggravates mitochondrial dysfunction by altering cristae structure,^[^ ^22^ ^]^ ATP synthesis,^[^ ^23^ ^]^ mitochondria dynamics,^[^ ^24^, ^25^ ^]^ and potentiating mitochondria fragmentation.^[^ ^26^ ^]^ Mutant Chchd10 binds to Chchd2 and TAR DNA‐binding protein 43 (TDP43) in response to cellular stress contributing to frontotemporal dementia–amyotrophic lateral sclerosis (FTD–ALS).^[^ ^26^, ^27^ ^]^ However, studies investigating the physiological roles of Chchd10 in maintaining mitochondria structure, oxidative phosphorylation, and antioxidant defense show inconsistent conclusions in different cell lines,^[^ ^23^, ^26^, ^28^, ^29^ ^]^ suggesting that Chchd10 may play distinct roles in different cell lineages. In addition to mitochondria, Chchd10 is also expressed in the nucleus and is involved in the transcriptional regulation of mitochondrial proteins.^[^ ^26^, ^30^ ^]^ Notably, recent studies reveal that Chchd10 is induced in fat depots in response to cold stimulation to activate brown adipose tissue (BAT) and mediates white adipose tissue (WAT) browning^[^ ^31^, ^32^ ^]^ while ablation of Chchd10 impairs lipolysis and suppresses uncoupling protein 1 (UCP‐1) expression, thus attenuating adaptive thermogenesis under fasting conditions.^[^ ^31^, ^32^ ^]^ Chchd10 is dramatically and persistently upregulated during brown adipocyte differentiation while it is transiently increased and then decreased during white adipocyte differentiation,^[^ ^31^ ^]^ suggesting Chchd10 may exert distinct functions in the early and late stages of white adipocyte differentiation. However, the role of Chchd10 in adipose tissue remodeling in response to energy surplus remains unclear.

In the present study, adipose tissue‐specific Chchd10 knockout (ATC10‐KO) mice and their relative Chchd10 gene wildtype (C10‐WT) littermates were subjected to STC or HFD feeding for short‐ or long‐term to explore the role of Chchd10 in adipocytes in response to acute and chronic conditions of excessive energy intake. The underlying mechanism of Chchd10 in regulating adipose tissue remodeling was determined using stromal vascular fraction (SVF)‐derived Chchd10 KO and WT adipocytes.

## 2 Results

### 2.1 Chchd10 Expression is Rapidly and Persistently Reduced in the WAT of HFD‐Fed Mice

**Figure 1:**
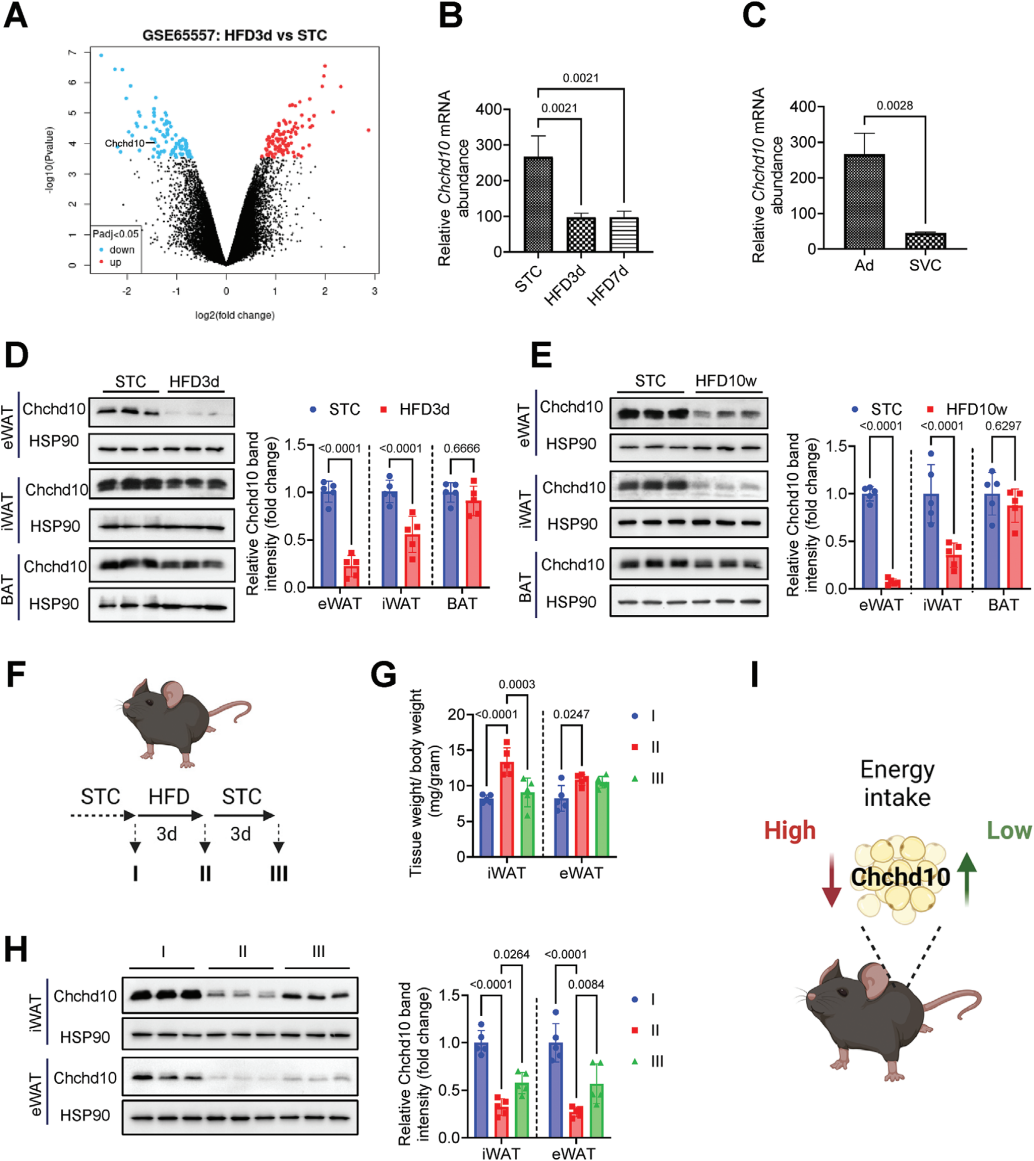
Chchd10 expression is rapidly and persistently reduced in the WAT of HFD‐fed mice. A–C) Analysis of the GEO dataset from GSE65557 provides RNA sequencing results of adipocytes isolated from eWAT of mice subjected to standard chow (STC) or high‐fat diet (HFD) feeding for 3 days or 7 days. A) Volcano plot showing the top significantly upregulated genes (red) and downregulated (blue) genes with 2‐fold changes in adipocytes of 3‐day HFD‐fed mice when compared to that of STC‐fed mice (*n* = 3). B) The mRNA abundance (log‐transformed values) of Chchd10 in adipocytes of mice as indicated (*n* = 3). C) The mRNA abundance of Chchd10 in adipocytes (Ad) and stromal vascular cells (SVC) of STC‐fed mice (*n* = 3). D) Representative immunoblots of the protein abundance of Chchd10 and HSP90 in eWAT, iWAT, and BAT of C57BL/6N mice fed with STC or HFD for 3 days. The right panel is the quantification of Chchd10 band intensity normalized with HSP90 (*n* = 5). E) Representative immunoblots of the protein abundance of Chchd10 and HSP90 in eWAT, iWAT, and BAT of C57BL/6N mice fed with STC or HFD for 10 weeks. The right panel is the quantification of Chchd10 band intensity normalized with HSP90 (*n* = 5). F–H) C57BL/6N mice were subjected to repeated 3 days of STC or HFD feeding as indicated (*n* = 5). F) Schematic diagram showing the feeding protocol and the classification of mouse groups. G) Mouse fat pad weight normalized with body weight (*n* = 5). H) Representative immunoblots of the protein abundance of Chchd10 and HSP90 in iWAT and eWAT of mice. The right panel is the quantification of Chchd10 band intensity normalized with HSP90 (*n* = 5). I) Summary of Chchd10 changes in WAT of mice under relatively high or low energy intake conditions, respectively. Data are presented as mean ± SD. Statistical significance was analyzed by Mann‐Whitney *U* test C, D, and E) or by one‐way ANOVA B, G, and H).

To identify the factors regulating adipose tissue homeostasis in response to HFD feeding, RNA sequencing datasets of white adipocytes isolated from short‐term HFD‐fed mice and STC‐fed mice (GSE65557) were compared, and Chchd10 was identified as one of the top downregulated genes (**Figure** **1**A). Consistently, there was approximately a 64% reduction of Chchd10 mRNA abundance in white adipocytes of 3 days or 7 days HFD‐fed mice compared to those of STC‐fed mice (Figure 1B). In adipose tissue, Chchd10 was mainly expressed in adipocyte (Ad) fraction rather than the stromal vascular cells (SVCs) (Figure 1C). The rapid reduction of Chchd10 protein abundance was further confirmed in mouse inguinal and epididymal WAT (iWAT and eWAT) after 3 days of HFD feeding, while Chchd10 expression in BAT was not altered (Figure 1D). In addition, sustained reduction of Chchd10 in iWAT and eWAT was observed in 10‐week long‐term diet‐induced obese (DIO) mouse model (Figure 1E). These findings implicated that Chchd10 expression is sensitive to the change of energy intake in WAT but not in BAT.

To determine the dynamic change of Chchd10 expression in response to energy intake, mice were subjected to a 3‐day STC and 3‐day HFD repeated feeding protocol (Figure 1F). Three days of HFD feeding significantly increased the relative weight of both iWAT and eWAT, while reverting back to STC reduced iWAT mass but not that of eWAT (Figure 1G), which is possibly due to the discrepancy of the two fat depots in heterogeneity, gene expression patterns, and metabolic activities.^[^ ^33^, ^34^, ^35^ ^]^ In both fat pads, Chchd10 expression was significantly reduced upon HFD feeding while recovered when re‐fed with STC (Figure 1H). Collectively, these findings suggested that Chchd10 may act as a metabolic sensor in white adipocytes in response to changes in energy intake, and its reduction upon excess energy intake is associated with an increase in fat mass (Figure 1I).

### 2.2 Adipose Tissue‐Specific Chchd10 Deficiency Enhances Adipogenesis Upon Excess Energy Intake While Protects Against Obesity and its Related Metabolic Disorders

To explore the function of Chchd10 in adipocytes, ATC10‐KO mice were generated using the Cre‐loxP system to delete exon 2 of Chchd10 gene and led to frameshift of the remaining exons (Figure S1A, Supporting Information). Chchd10 was specifically deleted in both WAT and BAT of ATC10‐KO mice without affecting its expression in other organs/tissues (Figure S1B, Supporting Information).

**Figure 2:**
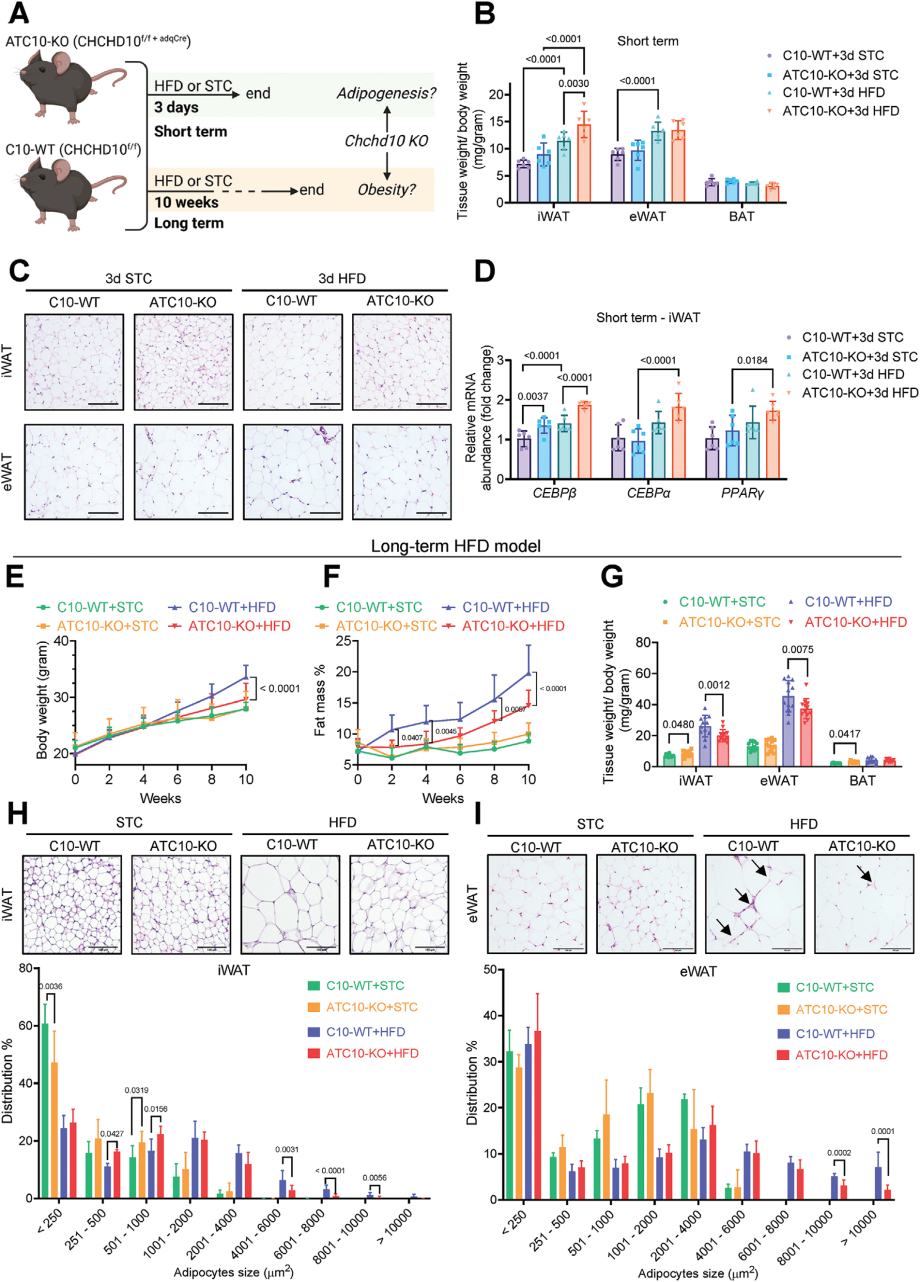
Adipose tissue‐specific Chchd10 deficiency enhances adipogenesis in response to excess energy intake while protects against obesity and its related metabolic disorders. A) Schematic diagram showing the short‐term and long‐term HFD or STC feeding protocols applied in adipose tissue‐specific Chchd10 knockout (ATC10‐KO) mice and their relative Chchd10‐wildtype (C10‐WT) littermates to determine the role of Chchd10 in adipose tissue (AT) adipogenesis and obesity, respectively. B–D) Six‐week‐old ATC10‐KO male mice and C10‐WT mice were subjected to HFD or STC feeding for 3 days (*n* = 6). B) Mouse fat pad weight normalized with body weight (*n* = 6). C) Representative images of H&E staining of mouse iWAT (upper) and eWAT (lower) (scale bar = 100 µm). D) The relative mRNA abundance of adipogenic markers (*CEBPβ, CEBPα*, and *PPARγ*) in iWAT of mice (*n* = 6). E–G) Six‐week‐old ATC10‐KO male mice and C10‐WT mice were subjected to HFD or STC feeding for 10 weeks (*n* = 12). E) Dynamic change of mouse body weight (n = 12). F) Dynamic change of mouse fat mass percentage (*n* = 12). (G) Mouse fat pad weight normalized with body weight (*n* = 12). H–I) Representative images of H&E staining of mouse G) iWAT and H) eWAT (scale bar = 100 µm). The lower panels are the distribution of adipocytes with different sizes in iWAT and eWAT, respectively (*n* = 8). Data are presented as mean ± SD. Statistical significance was analyzed by two‐way ANOVA B, D, E, F, G, H, and I).

To determine the role of Chchd10 in adipogenesis and the development of obesity, ATC10‐KO mice and their relative C10‐WT littermates were subjected to STC or HFD feeding for 3 days (short‐term model) or 10 weeks (long‐term model), respectively (**Figure** **2**A). In the short‐term model, HFD feeding significantly increased the relative weight of iWAT and eWAT but not BAT in C10‐WT mice (Figure 2B). Notably, the increase of iWAT weight was significantly higher in ATC10‐KO mice than that in C10‐WT mice (Figure 2B). Morphologically, there were more small size adipocytes in iWAT, but not in eWAT, of ATC10‐KO mice fed with either STC or HFD than those of the relative C10‐WT mice (Figure 2C). Consistently, the gene expression of CCAAT/enhancer‐binding protein beta (CEBPβ), an adipocyte differentiation marker, was significantly higher in the iWAT of ATC10 KO mice subjected to STC or HFD feeding when compared to that of the relative C10‐WT mice. Moreover, HFD feeding significantly induced the expression of two other adipogenic markers, CCAAT/enhancer‐binding protein alpha (CEBPα) and peroxisome proliferator‐activated receptor gamma (PPARγ), in ATC10‐KO mice but not in C10‐WT mice (Figure 2D). These findings implicated that Chchd10 deficiency potentiates adipogenesis in iWAT for lipid storage under short‐term HFD feeding.

In the long‐term model, no significant differences in body weight and fat mass gain were observed in STC‐fed C10‐WT and ATC10‐KO mice, while the HFD‐induced body weight and fat mass gain of ATC10‐KO mice were significantly attenuated compared to those of the relative C10‐WT mice throughout the feeding period (Figure 2E,F). The relative weight of iWAT and eWAT were also significantly lower in HFD‐fed ATC10‐KO mice when compared to those of the relative C10‐WT mice. Notably, there was a significant induction of iWAT and BAT weight in STC‐fed ATC10‐KO mice when compared to those of C10‐WT mice (Figure 2G). In iWAT of STC‐fed groups, the percentage of small adipocytes (size < 250 µm^2^) of ATC10‐KO was significantly lower, while the percentage of medium adipocytes (501 – 1000 µm^2^) was significantly higher than that of WT mice (Figure 2H). In contrast, in the HFD‐fed groups, the percentage of small to medium adipocytes (251 – 1000 µm^2^) of ATC10‐KO mice was significantly higher, while the percentage of large adipocytes (4001 – 10 000 µm^2^) was significantly lower than that of C10‐WT mice (Figure 2H). In eWAT, no significant difference in adipocyte distribution was observed in STC‐fed ATC10‐KO mice and C10‐WT mice but the percentage of large adipocytes (size > 8001 µm^2^) was significantly lower in HFD‐fed ATC10‐KO mice when compared to that of the relative C10‐WT mice, respectively (Figure 2I). Moreover, HFD‐induced immune cell accumulation, as indicated by the crown‐like structures, was reduced in eWAT of ATC10 KO mice compared to that of C10‐WT mice (Figure 2I). This evidence suggested that Chchd10 deficiency promotes lipid storage in iWAT over time while alleviating chronic HFD‐induced obesity by attenuating adipocyte hypertrophy in both iWAT and eWAT.

**Figure 3:**
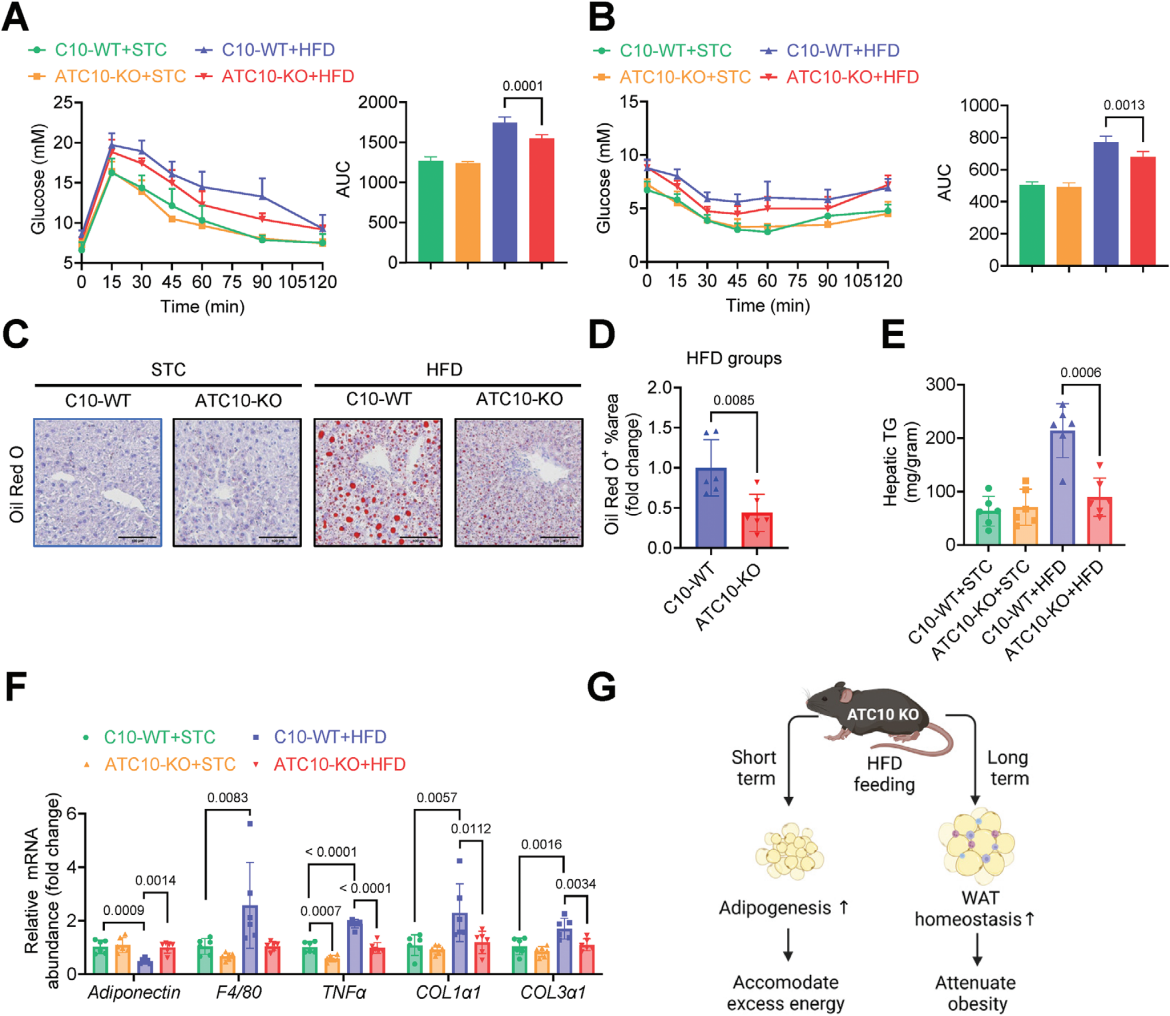
Chchd10 deficiency attenuates HFD‐induced adipose tissue dysfunction and metabolic disorders in mice. Six‐week‐old male ATC10‐KO mice and C10‐WT mice were subjected to HFD or STC feeding for 10 weeks (*n* = 12). A) Glucose tolerance test (left) and calculated areas under the curve (AUC, right) (*n* = 8). B) Insulin tolerance test (left) and calculated AUC (right) (*n* = 8). C) Representative images of Oil red O staining of mouse liver. D) The quantification of Oil red O positive area of HFD groups in C) (*n* = 6). E) The abundance of mouse hepatic triglyceride (TG) (*n* = 6). F) The mRNA abundance of adiponectin, inflammatory markers (F4/80 & TNFα), and fibrotic markers (Col1α1 and Col3α1) (*n* = 6). G) Summary of the phenotypes of ATC10‐KO mice compared to C10‐WT mice subjected to short‐term and long‐term HFD feeding models. Data are presented as mean ± SD. Statistical significance was analyzed by two‐way ANOVA A, B, E, and F) or Student's *t*‐test D).

Compared to C10‐WT mice, long‐term HFD‐fed ATC10‐KO mice displayed improved glucose and insulin tolerance (**Figure** **3**A,B) and attenuated hepatic steatosis (Figure 3C,D,E). As eWAT dysfunction is closely associated with metabolic complications,^[^ ^36^ ^]^ its function was further evaluated. HFD‐mediated reduced adiponectin expression in C10‐WT mice was significantly attenuated in ATC10‐KO mice. Furthermore, HFD‐induced macrophage infiltration as indicated by increased expression of F4/80, proinflammatory cytokine tumor necrosis factor alpha (TNFα), and fibrotic collagens (Col1α1 and Col3α1), which contribute to adipose tissue rigidity,^[^ ^2^ ^]^ were also significantly attenuated in ATC10‐KO mice (Figure 3F). These data implicated that Chchd10 deficiency protects against HFD‐induced WAT dysfunction associating with improved systemic metabolism in mice.

Taken together, Chchd10 deficiency promotes adipogenesis in iWAT to facilitate excessive lipid storage in response to short‐term energy surplus. In long‐term energy surplus, Chchd10 deficiency protects against diet‐induced obesity and metabolic disorders, possibly by improving WAT homeostasis (Figure 3G).

### 2.3 Chchd10 Deficiency Promotes Adipose Tissue Energy Expenditure under Obese Conditions

**Figure 4:**
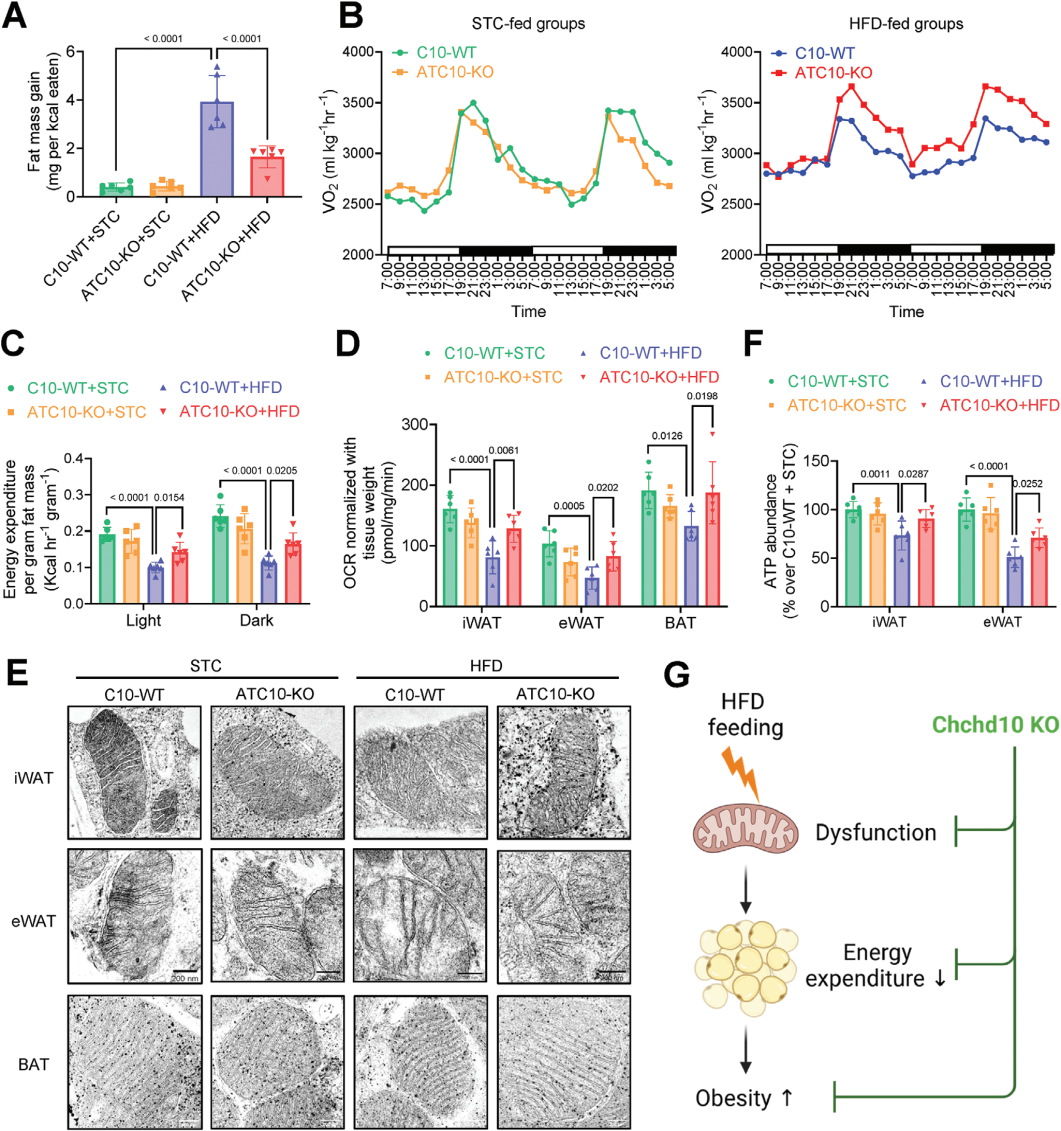
Chchd10 deficiency promotes adipose tissue energy expenditure under obese conditions. Six‐week‐old male ATC10‐KO mice and C10‐WT mice were subjected to STC or HFD feeding for 10 weeks. A) Fat mass gain (mg) per kcal consumed in mice (*n* = 6). B) Oxygen consumption (VO_2_) of mice of STC‐fed groups (left panel) and HFD‐fed groups (right panel) measured by comprehensive lab animal monitoring system (CLAMS, *n* = 6). C) Calculated energy expenditure of B) normalized with fat mass (*n* = 6). D) Oxygen consumption rate (OCR) of iWAT, eWAT, and BAT of mice normalized with tissue weight (*n* = 6). E) Representative images of mitochondrial structure in mouse iWAT, eWAT, and BAT as assessed by transmission electronic microscopy. F) ATP content in iWAT and eWAT of mice (*n* = 6). G) Chchd10 deficiency alleviates HFD‐induced mitochondrial dysfunction and impairment in adipose tissue energy expenditure, attenuating the development of obesity. Data are presented as mean ± SD. Statistical significance was analyzed by two‐way ANOVA A, C, D, and F).

Next, the whole‐body energy metabolism of mice was assessed. HFD feeding significantly increased the fat mass gain of C10‐WT mice (3.93 mg per Kcal intake) while that of ATC10‐KO mice (1.65 mg per Kcal intake) was ≈58% less (**Figure** **4**A). The whole‐body oxygen consumption rate (OCR) was comparable between STC‐fed C10‐WT and ATC10‐KO mice, while the OCR of HFD‐fed ATC10‐KO mice was significantly higher than that of C10‐WT mice (Figure 4B). When normalizing the energy expenditure with fat mass, STC‐fed ATC10‐KO and C10‐WT mice showed similar energy expenditure. On the contrary, HFD feeding significantly attenuated the energy expenditure of C10‐WT mice but not in ATC10‐KO mice (Figure 4C). Of note, no significant change in the energy expenditure was observed between the ATC10‐KO and C10‐WT mice subjected to either STC or HFD feeding when normalized with lean mass (Figure S2, Supporting Information). Consistent with the results of whole‐body energy expenditure, HFD feeding suppressed the OCR of isolated iWAT, eWAT, and BAT of C10‐WT mice, while ATC10 deficiency protected against this suppression (Figure 4D). These findings implicated that Chchd10 deficiency promotes energy expenditure of adipose tissue under obese conditions.

Given that Chchd10 is implicated in the maintenance of mitochondrial cristae structure and associated with the mitochondrial function of brown adipocytes,^[^ ^32^ ^]^ the morphologies of mitochondria in the three fat pads were assessed. The morphologies of mitochondria in iWAT, eWAT, and BAT were comparable between STC‐fed C10‐WT and ATC10‐KO mice. Notably, HFD feedingh reduced the mitochondrial cristae in eWAT but not in the other two fat pads of C10‐WT mice, while this reduction was alleviated in ATC10‐KO mice (Figure 4E). Furthermore, HFD feeding significantly impaired mitochondrial function in both iWAT and eWAT of C10‐WT mice as indicated by the decreased ATP production, which was alleviated in those of ATC10‐KO mice (Figure 4F).

Taken together, these findings indicated that Chchd10 deficiency enhances energy expenditure and protects against diet‐induced obesity, possibly through alleviating diet‐induced mitochondrial dysfunction in adipocytes (Figure 4G). Sustained Chchd10 reduction in response to long‐term HFD feeding may be an adaptive response to maintain adipose tissue homeostasis. Since Chchd10 abundance was not significantly changed in BAT after HFD‐feeding (Figure 1D,E), and no obvious alternation in the mitochondrial cristae structure (Figure 4E) and UCP‐1 expression (Figure S3, Supporting Information) was observed in Chchd10 deficient BAT, we therefore focused on the role of Chchd10 reduction in white adipocytes for the mechanistic studies.

### 2.4 Chchd10 Deficiency Promotes Adipogenesis, Improves Mitochondrial Function and GSH Metabolism in WAT

**Figure 5:**
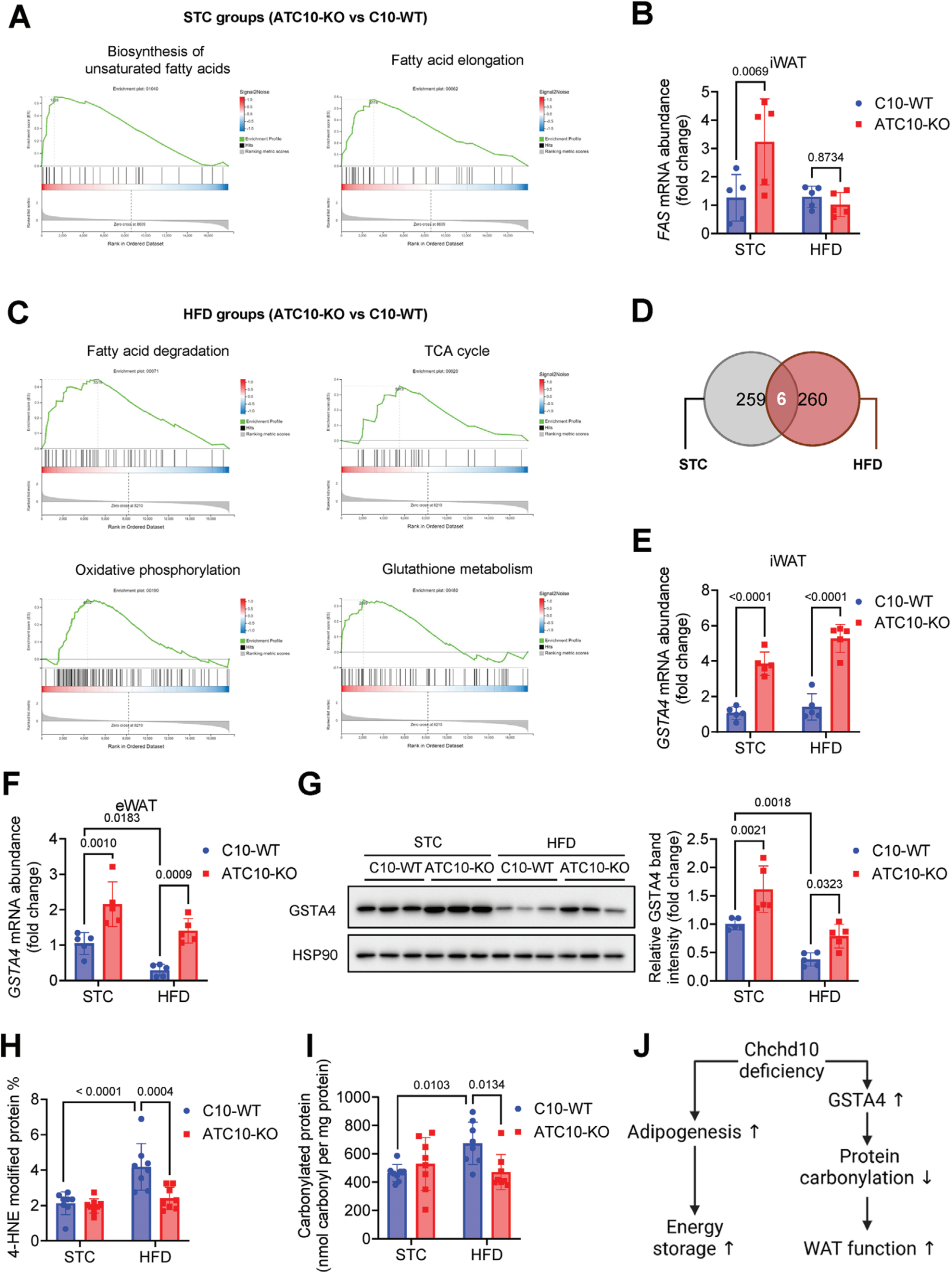
Chchd10 deficiency promotes adipogenesis and improves mitochondrial function and GSH metabolism in WAT. Six‐week‐old male ATC10‐KO mice and C10‐WT mice were subjected to STC or HFD feeding for 10 weeks. The mRNA extracted from mouse iWAT was subjected to RNA sequencing (RNA‐seq) analysis (*n* = 5). A) Gene set enrichment analysis of iWAT of ATC10‐KO mice compared to C10‐WT mice (*n* = 5, adjusted *p* < 0.05). B) The relative mRNA abundance of FAS in mouse iWAT (*n* = 5). C) Gene set enrichment analysis of iWAT of ATC10‐KO mice compared to C10‐WT mice (*n* = 5, adjusted *p* < 0.05). D) Venn diagram showing the intersection of differentially expressed genes in the two comparisons identified by RNA‐Seq. E,F) The relative mRNA abundance of GSTA4 in mouse E) iWAT and F) eWAT (*n* = 5). G) Representative immunoblots of the protein abundance of GSTA4 and HSP90 in mouse eWAT. The right panel is the quantification of GSTA4 band intensity normalized with HSP90 (*n* = 5). H,I) The abundance of H) 4‐HNE modified protein and I) carbonylated protein in mouse eWAT (*n* = 8). J) Potential pathways contributing to enhanced energy storage and attenuated protein carbonylation in Chchd10 deficient WAT. Data are presented as mean ± SD. Statistical significance was analyzed by two‐way ANOVA B, E, F, G, H, and I).

The underlying mechanisms of Chchd10 reduction in adipocytes to facilitate lipid storage and improve adipose tissue function in response to HFD feeding were then explored. Induction of iWAT mass in long‐term STC‐fed ATC10‐KO mice recapitulated the iWAT mass increase in short‐term HFD‐fed ATC10‐KO mice (Figure 2B,G). Under long‐term HFD feeding, iWAT shares a similar phenotype with eWAT as the weight gain and formation of large adipocytes were attenuated in both fat pads of ATC10 KO‐mice when compared to that of C10‐WT mice (Figure 2G,H,I). Hence, iWAT of the long‐term STC‐ and HFD‐fed mice were used for RNA sequencing and gene set enrichment analysis (GSEA). In the iWAT of STC‐fed ATC10‐KO mice, GSEA identified the upregulation of transcripts involved in the biosynthesis of unsaturated fatty acid and fatty acid elongation (**Figure** **5**A). A significant induction of mRNA expression of fatty acid synthase (FAS), the rate‐limiting enzyme of fatty acid synthesis, was validated in the iWAT of STC‐fed ATC10 KO mice when compared to that of C10‐WT mice (Figure 5B). This evidence aligns with our findings that Chchd10 deficiency facilitates adipogenesis in iWAT to accommodate excess energy intake.

In the iWAT of HFD‐fed ATC10‐KO mice, there was an upregulation of transcripts related to mitochondrial functions including fatty acid degradation, tricarboxylic acid cycle (TCA) cycle, and oxidative phosphorylation (Figure 5C). Besides, genes related to GSH metabolism that are responsible for antioxidative response^[^ ^37^ ^]^ were also upregulated in ATC10‐KO mice (Figure 5C). Among the six differentially expressed genes (UAP1, EIF4A2, GSTA4, Morf4l1b, ZFP949, CRTAC1) between ATC10‐KO mice and C10‐WT mice that fell in the intersection of STC‐fed and HFD‐fed conditions (Figure 5D), glutathione S‐transferase A4 (GSTA4) is a potent antioxidative enzyme that catalyzes the GSH‐mediated clearance of 4‐HNE.^[^ ^19^ ^]^ GSTA4 is downregulated in omental and subcutaneous fat depots of obese and insulin‐resistant individuals but not in obese and insulin‐sensitive individuals. The expression levels of GSTA4 in adipose tissue are also negatively correlated with homeostatic model assessment of insulin resistance (HOMA‐IR).^[^ ^19^ ^]^ GSTA4 deficiency leads to 4‐HNE accumulation and reduces the antioxidant capacity of mice.^[^ ^38^, ^39^ ^]^ These findings highlight the clinical relevance of GSTA4 downregulation and adipose tissue dysfunction and reinforce the essential role of GSTA4 in eliminating 4‐HNE and maintaining antioxidant capacity in adipose tissue. A significant upregulation of GSTA4 gene transcription was validated in the iWAT of ATC10‐KO mice subjected to either STC or HFD feeding (Figure 5E). As downregulation of GSTA4 in VAT of mice was found to increase 4‐HNE‐mediated protein carbonylation, oxidative stress, and mitochondrial dysfunction to exaggerate obesity and insulin resistance,^[^ ^19^ ^]^ the expression of GSTA4 and the abundance of 4‐HNE‐modified protein and carbonylated protein in eWAT were also determined. Under STC‐feeding, mRNA and protein levels of GSTA4 were significantly higher in eWAT of ATC10‐KO mice than that of C10‐WT mice. Moreover, HFD‐induced GSTA4 reduction (Figure 5F,G) and elevation of 4‐HNE modified protein and carbonylated protein in eWAT in C10‐WT mice (Figure 5H,I) were significantly alleviated in ATC10‐KO mice.

Taken together, these findings implicated that Chchd10 deficiency promotes adipogenesis to facilitate energy storage and upregulates GSTA4 expression to counteract 4‐HNE‐mediated protein carbonylation and improve adipocyte function (Figure 5J).

### 2.5 Chchd10 Deficiency Promotes Adipogenesis and Upregulates GSTA4 to Improve Adipocyte Function Under Insulin‐Resistant Conditions

**Figure 6:**
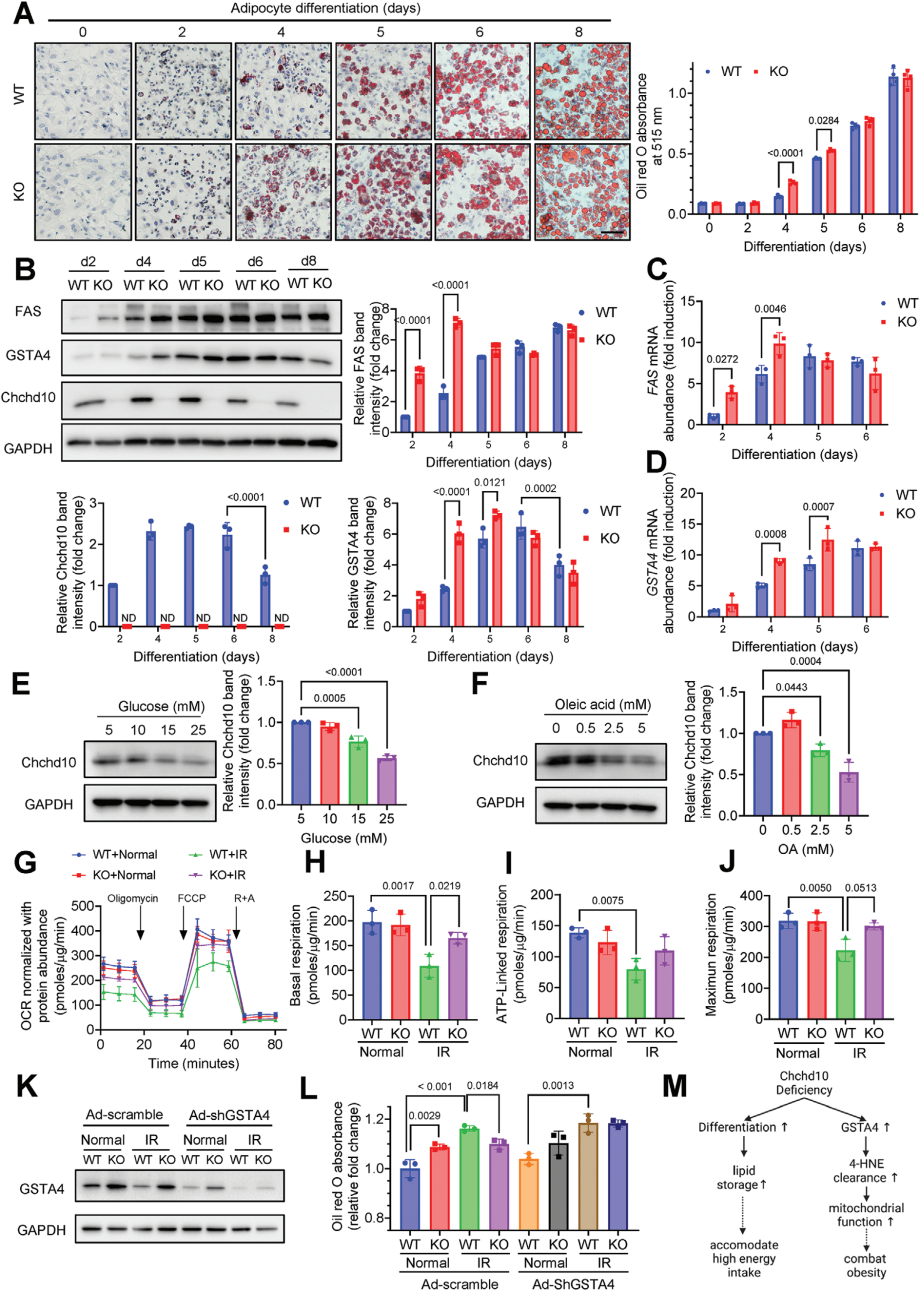
Chchd10 deficiency promotes adipogenesis and upregulates GSTA4 to improve adipocyte function under insulin‐resistant conditions. Stromal vascular fraction isolated from male C10‐WT mice and ATC10‐KO mice were subjected to in vitro differentiation to obtain wildtype (WT) or Chchd10 knockout (KO) white adipocytes, respectively. A) The representative images of Oil Red O staining of WT or Chchd10 KO white adipocytes during differentiation. The right panel is the quantification of neutral lipids contents (*n* = 4, scale bar = 100 µm). B) The representative immunoblots of the protein abundance of FAS, GSTA4, Chchd10, and GAPDH during white adipocyte differentiation. The lower and the right panels are the quantification of Chchd10, FAS, and GSTA4 band intensity normalized with GAPDH, respectively (*n* = 3). C,D) The relative mRNA abundance of C) FAS and D) GSTA4 during adipocyte differentiation (*n* = 3). E,F) The representative immunoblots of the protein abundance of Chchd10 and GAPDH in 4‐day‐differentiated adipocytes subjected to E) glucose and F) oleic acids treatment for 6 h. The right panels are the quantification of Chchd10 band intensity normalized with GAPDH (*n* = 3). G–J) 4‐day‐differentiated WT and Chchd10 KO adipocytes were subjected to an insulin‐resistance (IR) model or normal culture conditions for 16 h (*n* = 3). G) The oxygen consumption rate (OCR) of cells at basal condition or upon treatment of oligomycin (5µm), FCCP (0.3µm), and rotenone (2µm) plus antimycin A (2µm) (*n* = 3). H–J) Calculation of H) basal respiration, I) ATP‐linked respiration, and J) maximum respiration of cells in G) (*n* = 3). K,L) 2‐day‐differentiated WT and Chchd10 KO adipocytes were infected with adenovirus expressing scramble RNA (Ad‐scramble) or GSTA4 shRNA (Ad‐shGSTA4) for 48 h and subsequently subjected to IR model or normal culture condition for 16 h. (K) Representative immunoblots of the protein abundance of GSTA4 and GAPDH in the cells. L) The quantification of neutral lipid content in the cells as determined by Oil red O staining (*n* = 3). M) Schematic diagram illustrating the mechanism by which Chchd10 deficiency potentiates energy storage to accommodate excess energy intake and improves mitochondrial functions in adipocytes to combat obesity. Data are presented as mean ± SD. Statistical significance was analyzed by two‐way ANOVA A, B, C, D, H, I, J, L) or one‐way ANOVA E and F).

Next, we further evaluated whether Chchd10 deficiency promotes adipogenesis and the expression of GSTA4 in adipocytes. Since lipogenesis is a key event during in vitro adipocyte differentiation,^[^ ^40^, ^41^ ^]^ the lipid content and FAS expression were used as phenotypic and protein markers, respectively, to indicate adipogenesis in the subsequent study. The abundance of neutral lipids was significantly higher in Chchd10 KO adipocytes on or before day 5 of differentiation than that of the WT adipocytes, while this difference was diminished thereafter (**Figure** **6**A). Consistently, the elevated protein and mRNA expression of FAS in Chchd10 KO adipocytes was also diminished on and after day 5 of differentiation (Figure 6B,C). Similarly, the protein and mRNA expression of GSTA4 was significantly increased in Chchd10 KO adipocytes on and before day 5 of differentiation (Figure 6B,D) compared to that in the WT adipocytes. Notably, during adipocyte differentiation, the expression of Chchd10 was gradually increased from day 2 to day 6 but then reduced in 8‐day differentiated cells (Figure 6B). To further explore the cause of Chchd10 reduction in response to excess energy intake, the high Chchd10‐expressing adipocytes (4‐day‐differentiated) were stimulated with common energy sources, glucose and oleic acids (unsaturated fatty acids). The expression of Chchd10 was significantly reduced upon glucose and oleic acid challenges in a dose‐dependent manner (Figure 6E,F). Taken together, these findings implicated that excess energy intake (high glucose or FA consumption) triggers Chchd10 reduction in adipocytes to mediate adaptive response, including enhanced adipogenesis and GSTA4 to maintain adipose tissue homeostasis.

We then determined if the beneficial effect of Chchd10 deficiency in combating obesity and insulin resistance (IR) is mediated through upregulating GSTA4. The 4‐day‐differentiated Chchd10 KO adipocytes with higher GSTA4 expression and their relative WT adipocytes were subjected to an in vitro IR model (mimicking the HFD model) with TNFα supplementation and hypoxia^[^ ^42^ ^]^ or normal culture condition. No significant differences were observed in the respiration profile of Chchd10 KO and WT adipocytes cultured in a normal culture condition. However, IR model impaired basal respiration, ATP‐linked respiration, and maximum respiration capacity in WT adipocytes implicating mitochondrial dysfunction, which was significantly alleviated in Chchd10 KO adipocytes (Figure 6G,H,I,J). We further evaluated if the beneficial effect of Chchd10 deficiency in protecting against IR‐mediated mitochondrial dysfunction is GSTA4 dependent. Upon adenovirus‐mediated GSTA4 knockdown (Figure 6K), the effect of Chchd10 deficiency in alleviating IR model‐induced lipid accumulation was diminished (Figure 6L; Figure S4, Supporting Information). This evidence indicated that upregulated GSTA4 upon Chchd10 reduction improves mitochondrial functions and prevents excessive lipid accumulation in adipocytes under obese conditions.

Taken together, these findings implicated that in small adipocytes with relatively low lipid content, high energy intake‐induced Chchd10 reduction enhances lipogenesis to convert excessive energy into lipids for storage. On the other hand, Chchd10 reduction‐mediated upregulation of GSTA4 improves mitochondrial functions to prevent adipocyte hypertrophy and combat obesity (Figure 6M).

### 2.6 Chchd10 Deficiency Promotes Adipogenesis and GSTA4 Expression by Activating p62/Keap1/NRF2 Axis

**Figure 7:**
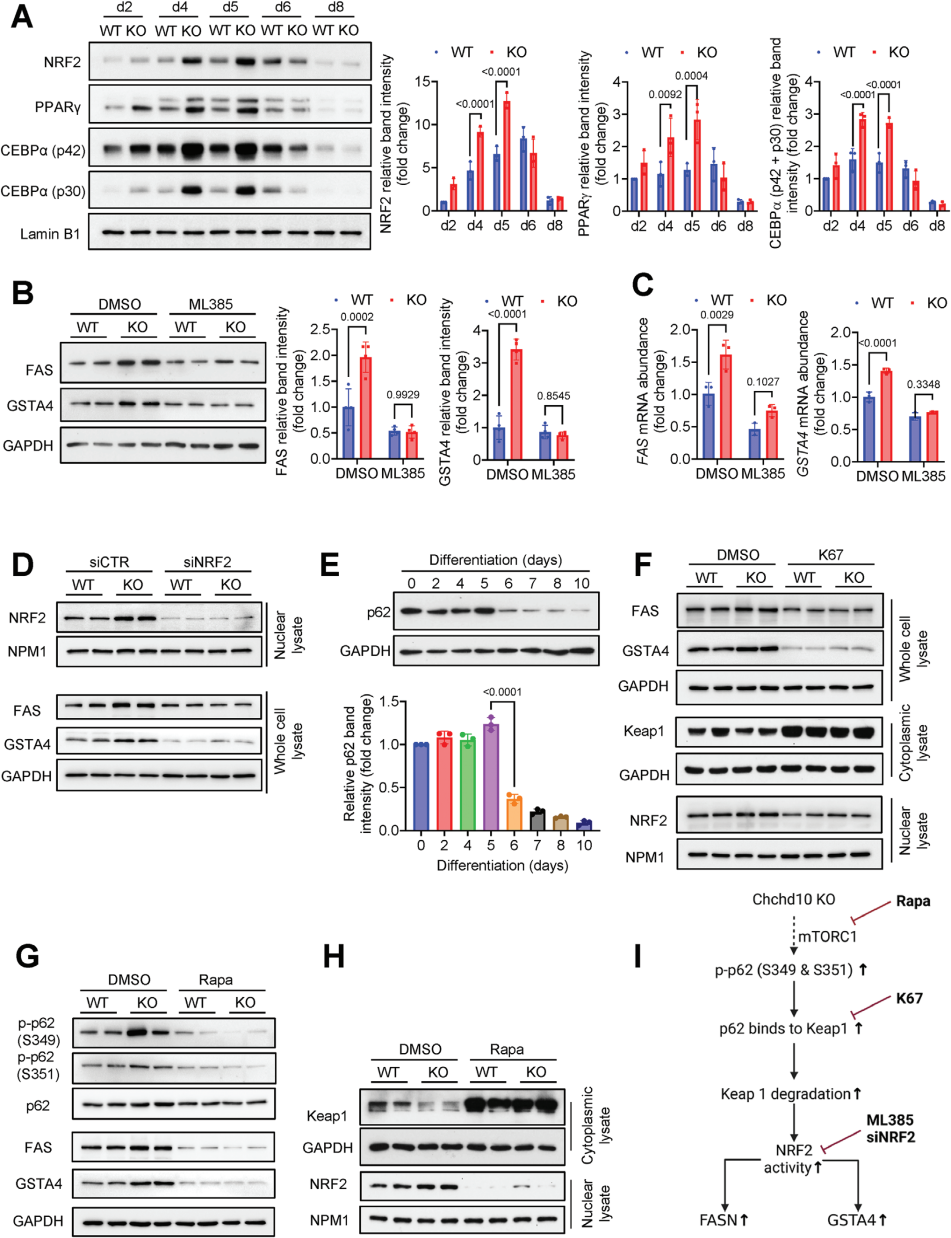
Chchd10 deficiency promotes adipogenesis and GSTA4 expression by activating p62/Keap1/NRF2 axis. Stromal vascular fraction isolated from male C10‐WT mice and ATC10‐KO mice were subjected to in vitro differentiation to obtain wildtype (WT) or Chchd10 knockout (KO) white adipocytes, respectively. A) Representative immunoblots of the nuclear abundance of NRF2, PPARγ, CEBPα (p42 and p30), and Lamin B1 during WT and Chchd10 KO white adipocyte differentiation. The right panels are the quantification of NRF2, PPARγ, and CEBPα (p42 + p30) band intensity normalized with Lamin B1 (*n* = 3). B,C) 2‐day‐differentiated WT and Chchd10 KO adipocytes were treated with ML395 (10µm) or its vehicle (DMSO) for 48 h. B) Representative immunoblots of the protein abundance of FAS, GSTA4, and GAPDH. The right panel is the quantification of FAS and GSTA4 band intensity normalized with GAPDH (*n* = 4). C) The relative mRNA abundance of FAS and GSTA4 (*n* = 3). D) 2‐day‐differentiated WT and Chchd10 KO adipocytes were treated with NRF2 siRNA (siNRF2) or controlled scramble siRNA (siCTR) for 48 h. Representative immunoblots of the protein abundance of NRF1 and nucleophosmin (NPM1) in the nuclear lysate and FAS, GSTA4, and GAPDH in the whole cell lysate. E) Representative immunoblots of the protein abundance of p62 and GAPDH during white adipocyte differentiation. The lower panel is the quantification of p62 band intensity normalized with GAPDH (*n* = 3). F) 2‐day‐differentiated WT and Chchd10 KO adipocytes were treated with K67 (10µm) or its vehicle (DMSO) for 48 h. Representative immunoblots of protein abundance of FAS, GSTA4, and GAPDH in the whole cell lysate, Keap1 and GAPDH in the cytoplasmic protein lysate, and NRF2 and NPM1 in the nuclear protein lysate, respectively. G,H) 2‐day‐differentiated WT and Chchd10 KO adipocytes were treated with rapamycin (Rapa, 200 nm) or vehicle (DMSO) for 48 h. G) Representative immunoblots of the protein abundance of p‐p62 (Ser351), p‐p62 (Ser349), p62, FAS, GSTA4, and GAPDH. H) Representative immunoblots of the protein abundance of Keap1 and GAPDH in cytoplasmic protein lysate and NRF2 and NPM1 in the nuclear protein lysate, respectively. I) Schematic diagram showing the mechanism by which Chchd10 deficiency activates p62/Keap1/NRF2 axis. Data are presented as mean ± SD. Statistical significance was analyzed by two‐way ANOVA A, B, and C) or one‐way ANOVA E).

The underlying mechanism of Chchd10 deficiency in promoting adipogenesis and GSTA4 expression was further investigated. Nuclear factor erythroid 2‐related factor 2 (NRF2) is a transcription factor regulating the expression of detoxification genes, including GSTA4.^[^ ^43^ ^]^ NRF2 also regulates adipogenesis by enhancing the expression of adipogenic initiators, including CEBPβ, CEBPα, and PPARγ.^[^ ^44^, ^45^ ^]^ The nuclear abundance of NRF2, PPARγ, and CEBPα was significantly higher in Chchd10 KO adipocytes within the first 5 days of differentiation but diminished thereafter (**Figure** **7**A). Hence, Chchd10 deficiency may upregulate adipogenesis and GSTA4 by enhancing NRF2 activity. To confirm this, the WT and Chchd10‐deficient adipocytes were treated with either NRF2 selective inhibitor ML385^[^ ^46^ ^]^ or its vehicle DMSO. The Chchd10 deficiency‐mediated upregulation of FAS and GSTA4 was attenuated upon NRF2 inhibition (Figure 7B,C). Consistently, FAS and GSTA4 upregulation in Chchd10‐deficient adipocytes were also abolished upon silencing of the NRF2 gene (Figure 7D; Figure S5A, Supporting Information). These data indicated that Chchd10 deficiency induces adipogenesis and GSTA4 expression through promoting NRF2 activity.

Next, we explored why Chchd10 deficiency only promotes NRF2 activity within the first 5 days of adipocyte differentiation. Activation of NRF2 in the nucleus requires dissociation from its negative regulator, Kelch‐like ECH‐associated protein 1 (Keap1), in the cytoplasm. p62 (also known as sequestosome1) is a multifunctional scaffold protein that can compete with NRF2 for binding to Keap1, potentiating the degradation of Keap1 and allowing the nuclear accumulation of NRF2.^[^ ^47^ ^]^ In turn, NRF2 can induce p62 expression, thus establishing a positive feedback loop for the p62/Keap1/NRF2 axis to perpetuate the antioxidative response.^[^ ^48^ ^]^ During the adipocyte differentiation, the consistent expression of p62 within the first 5 days of differentiation was strikingly reduced thereafter (Figure 7E). By blocking the Keap1 binding domain (KIR) of p62 with a p62‐specific inhibitor K67,^[^ ^49^, ^50^ ^]^ the increased FAS and GSTA4 expression, reduced cytoplasmic Keap1 abundance, and enhanced nuclear NRF2 accumulation in Chchd10 KO adipocytes were all abolished (Figure 7F; Figure S5B, Supporting Information). This evidence suggested that Chchd10 deficiency‐induced NRF2 activation depends on the enhanced activity of p62.

The phosphorylation of p62 at Ser351 or S349 by the mammalian target of rapamycin complex 1 (mTORC1) was reported to enhance its binding activity to Keap1 to promote Keap1 degradation.^[^ ^47^ ^]^ Upon inhibition of mTOR kinase by rapamycin (Rapa), the enhanced phosphorylation of p62 (Ser 351 and S349) (Figure 7G; Figure S5C, Supporting Information), the reduced Keap1 abundance, the increased nuclear NRF2 accumulation (Figure 7H; Figure S5D, Supporting Information), and the NRF2‐mediated upregulation of total p62, FAS, and GSTA4 (Figure 7G; Figure S5C, Supporting Information) in Chchd10 KO adipocytes were all abolished. These findings indicated that Chchd10 deficiency promotes adipogenesis and GSTA4 expression in adipocytes by activating the p62/Keap1/NRF2 axis in a mTORC1‐dependent manner (Figure 7I).

### 2.7 Chchd10 Deficiency Enhances TDP43‐Mediated Raptor mRNA Stabilization Thus Promoting the mTORC1‐Mediated p62/Keap1/NRF2 Activation

**Figure 8:**
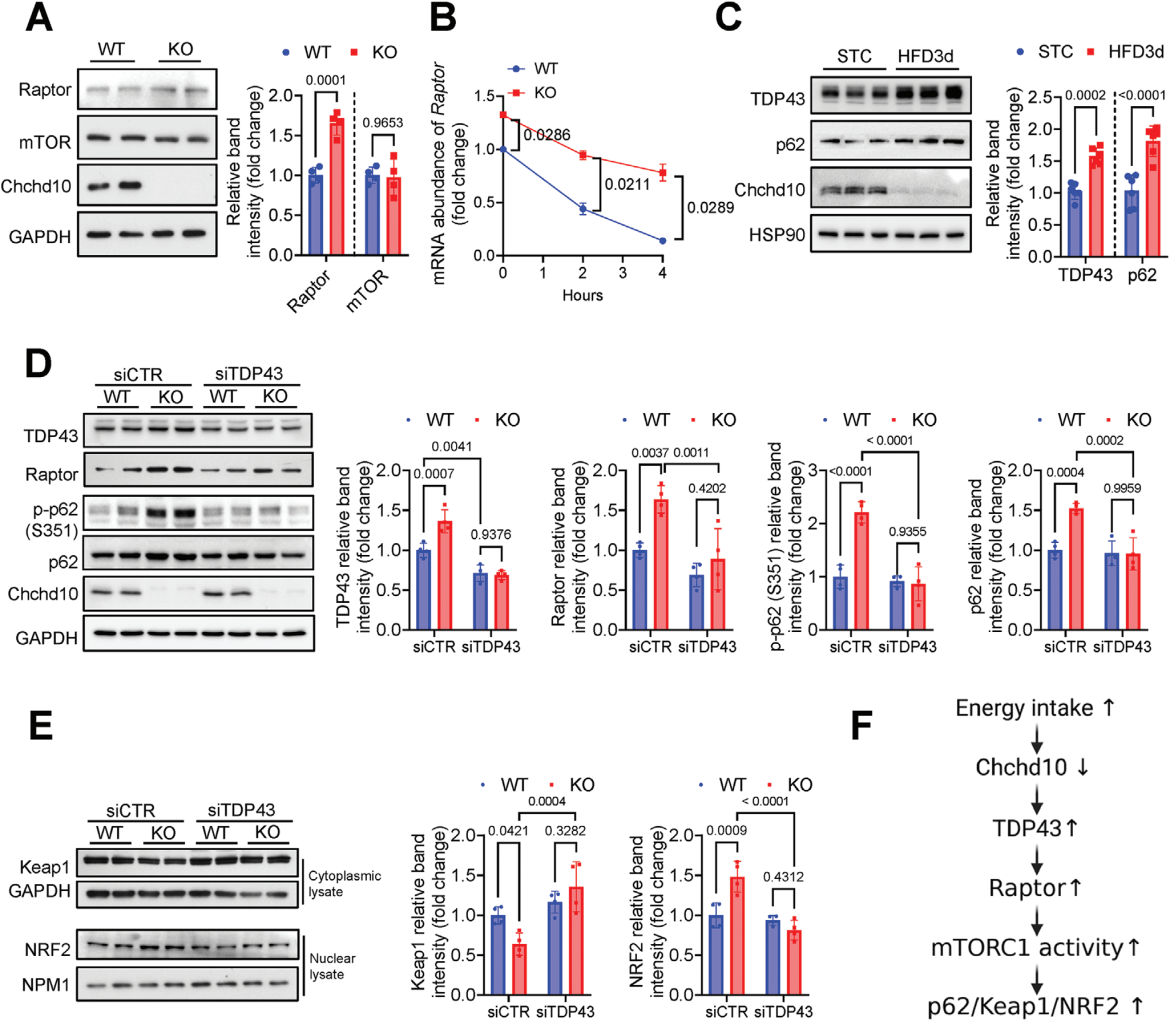
Chchd10 deficiency promotes TDP43‐mediated Raptor mRNA stabilization thus promoting the mTORC1‐mediated p62/Keap1/NRF2 activation. A) Representative immunoblots of the protein abundance of Raptor, mTOR, Chchd10, and GAPDH in 4‐day‐differentiated WT and Chchd10 KO adipocytes. The right panel is the quantification of Raptor and mTOR band intensity normalized with GAPDH (*n* = 4). B) The relative mRNA abundance of Raptor in 4‐day‐differentiated WT and Chchd10 KO adipocytes treated with actinomycin D for 0, 2, and 4 h (*n* = 3). C) Representative immunoblots of the protein abundance of TDP43, p62, Chchd10, and HSP90 in eWAT of WT mice fed with STC or HFD for 3 days. The right panel is the quantification of TDP43 and p62 band intensity normalized with HSP90 (*n* = 6). D,E) 2‐day‐differentiated WT and Chchd10 KO adipocytes were treated with TDP43 siRNA (siTDP43) or controlled scramble siRNA (siCTR) for 48 h. D) Representative immunoblots of the protein abundance of TDP43, Raptor, p‐p62 (S351), p62, Chchd10, and GAPDH. The right panel is the quantification of band intensity of TDP43, Raptor, p‐p62 (S351), and p62 normalized with GAPDH (*n* = 4). E) Representative immunoblots of the protein abundance of the Keap1 and GAPDH in the cytoplasmic protein lysate and NRF2 and NPM1 in the nuclear protein lysate, respectively. The right panel is the quantification of Keap1 band intensity normalized with GAPDH and NRF2 band intensity normalized with NPM1, respectively (*n* = 4). F) Schematic diagram showing the mechanism by which Chchd10 reduction upon excess energy intake promotes TDP43 mediated Raptor stabilization and the subsequent upregulation of mTORC1 activity and p62/Keap1/NRF2 axis. Data are presented as mean ± SD. Statistical significance was analyzed by Mann‐Whitney *U* test A, B and C) or two‐way ANOVA D and E).

The regulatory‐associated protein of mTOR (Raptor) is a critical regulator of mTORC1 activity. Raptor deficiency has been found to decrease p62 phosphorylation^[^ ^47^ ^]^ and impair adipogenesis.^[^ ^51^ ^]^ In this study, the protein expression of Raptor but not mTOR was significantly increased in Chchd10 KO adipocytes (**Figure** **8**A). Given that TDP43, a Chchd10 binding partner^[^ ^26^ ^]^ accumulated in the nucleus, was shown to stabilize Raptor mRNA, thus promoting mTORC1 lysosome localization and activation,^[^ ^52^ ^]^ we hypothesized that Chchd10 deficiency enhances TDP43‐mediated Raptor mRNA stabilization, hence activating mTORC1‐dependent p62/Keap1/NRF2 axis. With the treatment of a gene transcription inhibitor actinomycin D (ActD), the mRNA abundance of Raptor was significantly higher in Chchd10 KO adipocytes than in WT adipocytes (Figure 8B), implicating the stability of Raptor mRNA is higher in Chchd10 KO adipocytes.

Furthermore, the expression of TDP43 and p62 was significantly higher in the eWAT of 3‐day HFD‐fed mice, which was accompanied by Chchd10 reduction (Figure 8C). Hence, we postulated that Chchd10 reduction induces TDP43 expression, thus promoting Raptor/p62/Keap1/NRF2 axis activation. In adipocytes with siRNA‐mediated TDP43 knockdown (Figure 8D), the induction of Raptor, p‐p62 (S351), and total p62 expression (Figure 8D), the reduction of cytoplasmic Keap1 abundance, and the induction of nuclear NRF2 accumulation (Figure 8E) in Chchd10 KO adipocytes were all abolished. These findings implicated that increased TDP43 plays a key role in modulating Raptor upregulation and the subsequent p62/Keap1/NRF2 signaling in Chchd10‐deficient adipocytes. Taken together, reduction of Chchd10 enhances TDP43 expression in adipocytes, thus enhancing Raptor mRNA stability and the subsequent activation of the mTORC1‐mediated p62/Keap1/NRF2 axis (Figure 8F).

### 2.8 Abdominal Adipose Tissue‐Specific Chchd10 Overexpression Reduces Adiposity in STC‐Fed Mice While Exaggerates Obesity in HFD‐Fed Mice

To further confirm the role of Chchd10 in modulating adipogenesis and GSTA4 expression, Chchd10 was overexpressed in abdominal adipose tissue of C57BL/6N mice by intraperitoneal injection of AAV‐overexpressing Chchd10 (Rec2C10) or control AAV (Rec2Ctrl) followed by 4 weeks of HFD or STC feeding (Figure S6A, Supporting Information). Overexpression of Chchd10 in abdominal adipose tissues, including eWAT, perirenal WAT (periWAT), and mesenteric WAT (mesWAT), but not in peripheral adipose tissue (iWAT and BAT) or other organs or tissues (heart, liver, and muscle), was confirmed (Figure S6B, Supporting Information). At the endpoint, in STC‐fed groups, adipose tissue‐specific Chchd10 overexpressing (ATC10‐OE) mice exhibited a significant reduction in body weight compared to their relative control (Ctrl) mice. In contrast, in HFD‐fed groups, ATC10‐OE provoked diet‐induced body weight gain (Figure S6C, Supporting Information). Moreover, the relative weights of abdominal fat depots (eWAT, periWAT, and mesWAT) in STC‐fed AdC10 OE mice were lower than those of their relative Ctrl mice, whereas the opposite phenotype was observed under HFD‐fed condition (Figure S6D, Supporting Information). Furthermore, the expression of GSTA4 in eWAT was reduced in ATC10‐OE mice under both STC‐ and HFD‐fed conditions (Figure S6E, Supporting Information).

In 3T3L1 cell‐derived adipocytes, the expression of FAS, GSTA4, TDP43, and p62 and the nuclear abundance of NRF2 and PPARγ were reduced upon Chchd10 overexpression while the cytoplasmic abundance of Keap1 was increased (Figure S6F, Supporting Information). Taken together, the findings of fat pad weight gain and GSTA4 expression in ATC10‐OE mice are opposite to the phenotypes of ATC10‐KO mice subjected to a long‐term feeding model, which further confirms that Chchd10 reduction upon HFD feeding protects against diet‐induced adipose tissue dysfunction and obesity.

### 2.9 Tamoxifen‐Induced Conditional Adipose Tissue‐Specific Chchd10 Knockout Decelerates the Progression of Diet‐Induced Obesity in Mice

In light of the above findings demonstrating the beneficial effects of adipose tissue Chchd10 deficiency in attenuating diet‐induced obesity, we further investigated the potential effect of Chchd10 suppression during the development of obesity. Tamoxifen‐inducible adipose tissue‐specific Chchd10 knockout mice (iATC10‐KO) and their relative control mice were subjected to tamoxifen‐induced deletion of AT‐Chchd10 after 4 weeks of HFD induction followed by an additional 4 weeks of HFD feeding (Figure S7A, Supporting Information). The tamoxifen‐induced knockout of Chchd10 in adipose tissues of iATC10‐KO mice was confirmed at the endpoint (Figure S7B, Supporting Information). Although tamoxifen administration itself led to body weight loss and fat mass reduction in the first two weeks of injections,^[^ ^53^ ^]^ the gain of body weight and fat mass percentage upon HFD feeding was attenuated in iATC10‐KO mice when compared to the control mice (Figure S7C,D, Supporting Information). There was a significant reduction of iWAT, eWAT, and mesWAT in iATC10‐KO mice compared to the control mice (Figure S7E, Supporting Information). In addition, the expression of TDP43 and GSTA4 was significantly increased in eWAT of iATC10‐KO mice (Figure S7F, Supporting Information), indicating the activation of TDP43/Raptor/p62/Keap1/NRF2 axis in adipose tissue upon Chchd10 knockout. These findings reinforce the beneficial effect of Chchd10 reduction in adipose tissue at the early stage of obesity in maintaining adipose tissue homeostasis.

## 3 Discussion

**Figure 9:**
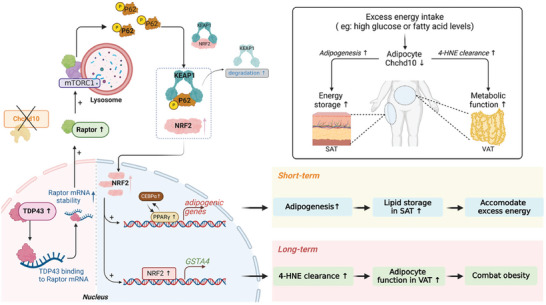
Summary of the effect of Chchd10 reduction in adipocytes in response to excess energy intake. In response to excess energy intake with high glucose and fatty acid levels, Chchd10 expression is rapidly and persistently reduced in white adipocytes. In short term, reduction of Chchd10 enhances adipogenesis predominantly in subcutaneous adipose tissue (SAT) to potentiate lipid storage, thus accommodating the excess energy intake. In the long term, Chchd10 reduction helps maintain the normal function of adipocytes, mainly in visceral adipose tissue (VAT), by promoting 4‐HNE clearance. Mechanistically, reduction of Chchd10 promotes TDP43 expression, stabilizing Raptor mRNA thus promoting Raptor protein synthesis. Increased Raptor promotes the localization of the mTORC1 complex on the lysosome and enhances the mTORC1‐mediated phosphorylation of p62. p‐p62 potentiates Keap1 degradation and enhances the dissociation of the Keap1‐NRF2 complex. Increased free NRF2 enters the nucleus. On one hand, NRF2 promotes the activity of PPARγ and CEBPα, thus promoting adipogenesis. On the other hand, NRF2 itself upregulates the expression of the antioxidative enzyme GSTA4. When faced with lipid peroxidation, enhanced GSTA4 catalyzes the GSH‐mediated 4‐HNE clearance, preventing the 4‐HNE‐mediated protein carbonylation, thereby protecting adipocyte function. Overall, reduction of Chchd10 enhances adipogenesis and the anti‐oxidant capacity of adipocytes by activating the TDP43/Raptor/p62/Keap1/NRF2 axis, which modulates WAT remodeling and maintains WAT homeostasis in response to excess energy intake.

Impaired adipogenesis and redox imbalance are hallmarks of disruption of adipose tissue homeostasis, contributing to obesity and metabolic disorders. In the present study, we identified the novel function of a mitochondrial protein Chchd10 which acts as a metabolic sensor that rapidly reduced in adipocytes in response to excess energy intake to facilitate adipose tissue remodeling and maintain adipose tissue homeostasis. On one hand, Chchd10 reduction potentiates adipogenesis predominantly in SAT to accommodate short‐term energy surplus. On the other hand, Chchd10 reduction upregulates GSTA4 which protects adipocytes mainly in VAT from 4‐HNE‐induced protein carbonylation and the subsequent adipocyte dysfunction under long‐term energy surplus. Mechanistically, Chchd10 reduction initiates the adaptive response by activating the TDP43/Raptor/p62/Keap1/NRF2 axis (**Figure** **9**). As obesity progresses, the loss of p62 in adipocytes with relatively high lipid contents impairs the Chchd10‐mediated adaptive mechanism, which eventually potentiates the development of obesity and its related metabolic disorders.

In previous studies, Chchd10 induction in response to cold exposure was shown to potentiate lipolysis, thus promoting BAT activation and WAT browning for energy expenditure.^[^ ^31^, ^32^ ^]^ In contrast, the present study revealed that Chchd10 reduction in WAT in response to excess energy intake promotes adipogenesis and redox balance, thus enhancing energy storage and maintaining adipose tissue homeostasis. Moreover, the expression of Chchd10 in WAT is rapidly decreased or increased in response to high‐ or low‐calorie diets, respectively. High glucose and fatty acid levels are the energy sources that stimulate the reduction of Chchd10 in white adipocytes. These findings collectively render the notion that Chchd10 acts as a metabolic sensor modulating energy expenditure and storage in adipose tissue. Mechanistically, Chchd10 reduction promotes the expression of the master regulators of adipogenesis (CEBPβ, PPARγ, and CEBPα) and the antioxidative enzyme GSTA4 through activating NRF2. Although studies of NRF2 in adipose tissue show conflicting results^[^ ^54^ ^]^ as both inhibition and activation of NRF2 have been shown to suppress obesity and metabolic diseases, the present study reinforces the importance of Chchd10 reduction‐mediated NRF2 activation as an adaptive response in adipocytes in combating obesity and its related metabolic disorders. In turn, the presence of Chchd10 in adipocytes under physiological conditions may restrict the NRF2‐mediated adipogenesis to control the adiposity in a healthy state. Apart from this, although Chchd10 is reported to be a component of the mitochondrial cristae organizing system (MICOS),^[^ ^55^ ^]^ the present study showed that Chchd10 is not involved in the mitochondrial cristae formation in adipocytes. This finding recapitulates the recent reports showing that Chchd10 is not a component of the MICOS in certain cell types, such as HEK293 cells and human fibroblasts.^[^ ^23^, ^25^, ^29^ ^]^ Moreover, we showed that Chchd10 deficiency did not alter the oxidative phosphorylation in adipocytes as observed in other cell lines.^[^ ^25^, ^26^, ^28^ ^]^ This evidence further implicated that adipocyte Chchd10 predominantly participates in regulating energy metabolism rather than mitochondrial functions.

Emerging evidence demonstrated that p62 plays a crucial role in obesity. p62‐deficient mice developed obesity, insulin resistance, and fatty liver spontaneously in their later lives.^[^ ^56^, ^57^ ^]^ p62 was shown to attenuate adipogenesis by inhibiting ERK signaling and macrophage inflammation in WAT while potentiating thermogenesis in BAT.^[^ ^58^ ^]^ Meanwhile, p62 is essential for maintaining leptin sensitivity in the brain to control food intake.^[^ ^59^ ^]^ Our study, for the first time, demonstrated that p62 is critical for modulating the Chchd10 reduction‐mediated adaptive response in white adipocytes by activating NRF2 activity. During SVF‐derived adipocyte differentiation, p62 is constantly expressed within the first 5 days while dramatically decreased thereafter. Since lipid accumulation is gradually increased during in vitro adipocyte differentiation, we postulated that p62 is reduced in adipocytes reaching a lipid storage threshold. Thus, it is possible that the loss of p62 in the hypertrophic adipocytes initiates the disruption of adipose tissue homeostasis, which is in line with the fact that adipose tissues from obese patients with T2DM and HFD‐fed mice have reduced p62 protein levels^[^ ^60^, ^61^ ^]^ and the presence of hypertrophic adipocytes exaggerates obesity.^[^ ^2^ ^]^ The underlying mechanisms that trigger the loss of p62 during adipocyte expansion warrant further investigations. On top of the mechanisms that p62 promotes NRF2 activity through potentiating Keap1 degradation,^[^ ^47^ ^]^ we showed that the phosphorylation of p62 in modulating the Keap1/NRF2 axis in adipocytes is mTORC1 dependent, which has been reported to enhance the binding affinity between p62 and Keap1.^[^ ^47^ ^]^ Given that p62 is a targeted gene of NRF2^[^ ^48^ ^]^ and p62 can activate mTOR under amino acid‐rich conditions,^[^ ^62^ ^]^ the formation of the positive feedback loop of mTOR‐p62‐NRF2 may further potentiate the Chchd10 reduction‐induced adaptive response in adipocytes in response to excess energy intake. Collectively, the present findings identify p62 as a key modulator of the Chchd10‐reduction mediated adaptive response and its reduction in hypertrophic adipocytes may lead to the conversion of flexible adipose tissue to rigid dysfunctional adipose tissue during the development of obesity.

TDP‐43 is a nuclear protein of the heterogeneous ribonucleoprotein (hnRNP) family, playing a major role in regulating RNA splicing, stability, and transport.^[^ ^63^ ^]^ The Chchd10 mutant S59L disrupts synaptic integrity by promoting the aggregation and mislocalization of nuclear TDP43 to the cytoplasm and mitochondria of neurons.^[^ ^26^ ^]^ Conversely, endogenous Chchd10 was proposed to retrogradely signal to the nucleus by binding to TDP43 and preventing its nuclear export in response to mitochondrial stress.^[^ ^26^ ^]^ In the present study, Chchd10 deficiency enhanced TDP43 expression in adipocytes, implying that Chchd10 is a negative regulator of TDP43 expression. The underlying mechanism warrants further investigation. Moreover, we found that increased TDP43 enhances the Raptor mRNA stability, thereby promoting mTORC1 activity to activate the p62/Keap1/NRF2 axis. Besides, the upregulation of TDP43 in promoting mTORC1 activity and the positive role of mTORC1 in modulating lipid synthesis^[^ ^64^ ^]^ at least partially contribute to the metabolic phenotype observed in TDP43 gene‐manipulated mice.^[^ ^65^, ^66^ ^]^ Briefly, postnatal deletion of TDP43 results in remarkable fat loss,^[^ ^65^ ^]^ while TDP43 overexpression leads to significant fat mass gain.^[^ ^66^ ^]^ Taken together, the present findings broaden the interplay between Chchd10 and TDP43 in adipocytes and illustrate the regulation of TDP43 on mTORC1‐mediated signaling pathways.

Clinical studies showed that peripheral obese individuals are more metabolic healthy,^[^ ^67^ ^]^ while central obesity is closely associated with metabolic complications,^[^ ^68^, ^69^ ^]^ suggesting that increased accumulation of SAT is more beneficial to health than accumulating VAT. Current anti‐obesity strategies, such as lifestyle interventions, medications, and bariatric surgery, mostly work by reducing overall adiposity but not targeting a specific adipose depot. These approaches are often limited by poor compliance, adverse side effects (eg., gastrointestinal symptoms and nutritional deficiencies), and a failure to combat weight regain effectively.^[^ ^70^ ^]^ In the present study, reduction of Chchd10 upon excess energy intake promotes adipogenesis in SAT in short‐term HFD feeding while protects against overall fat mass gain and metabolic disorders after long‐term HFD feeding by inducing GSTA4. These data indicate that enhanced lipid storage in newly formed adipocytes in SAT is the first step of the Chchd10 reduction‐mediated protective mechanism. The increased adipocytes in SAT but not VAT in Chchd10‐deficient mice may be due to the higher number and adipogenic capacity of adipocyte progenitor cells in SAT than in VAT.^[^ ^71^, ^72^, ^73^ ^]^ Among obese individuals with similar weight gain (≈6%), those with a significant increase in lipogenesis in SAT were more metabolic healthy and resistant to the development of cardiometabolic diseases compared to those who had more lipid accumulation in VAT.^[^ ^74^ ^]^ On the other hand, upregulated GSTA4 in adipocytes with low Chchd10 expression in both SAT and VAT plays a key role in long‐term protection against HFD induction. Particularly in VAT, which is closely associated with the metabolic outcome,^[^ ^4^, ^5^ ^]^ Chchd10 reduction‐mediated GSTA4 elevation prevents 4‐HNE‐mediated protein carbonylation. This attenuates mitochondrial dysfunction in adipocytes and alleviates adipokine dysregulation, inflammation, and adipose tissue fibrosis, thus enhancing adipose tissue energy expenditure and whole‐body metabolism, thereby alleviating obesity. Notably, although GSTA4 overexpression in adipose tissue of HFD‐induced mice has been shown to promote 4‐HNE excretion and reduce carbonylation adducts, the glucose tolerance and insulin sensitivity of the mice are not improved. It is suggested that the overactive excretion of glutathionyl lipid aldehydes results in increased exposure of tissue‐resident macrophages to these inflammatory metabolites leading to insulin resistance.^[^ ^75^ ^]^ Though Chchd10 KO mice do not show the neuromuscular degenerative phenotypes observed in patients with Chchd10 mutations,^[^ ^76^ ^]^ Chchd2 and Chchd10 double KO mice exhibit cardiomyopathy and mitochondrial integrated stress response in the heart.^[^ ^77^ ^]^ Thus, targeting Chchd10 may not contribute to neurodegenerative diseases but may potentially cause adverse effects in other organs. Further investigations on the safety of targeting Chchd10 for treating obesity are warranted.

In summary, the present study uncovers the novel role of Chchd10 as a metabolic sensor in modulating adipose tissue homeostasis. Through activating the TDP43/Raptor/p62/Keap1/NRF2 axis, the reduction of Chchd10 in response to energy surplus awakens the adaptive response, which potentiates adipogenesis and GSTA4‐mediated 4‐HNE clearance to combat obesity. In turn, the presence of Chchd10 in adipocytes may help govern adipogenesis under a healthy state. Notably, the loss of p62 during lipid accumulation in adipocytes is a potential factor disrupting Chchd10‐mediated adipose tissue homeostasis.

## 4 Experimental Section

### Animals and Experimental Models

To obtain ATC10‐KO mice and relative C10‐WT mice, Chchd10^flox/flox^ (Chchd10^f/f^) mice with C57BL/6N background were crossed with Adipoq‐Cre mice (the Jackson Laboratory, Strain #:010803). To obtain inducible adipose tissue‐specific Chchd10 knockout (iATC10‐KO) mice and relative control mice, Chchd10^f/f^ mice were crossed with Adipoq‐iCreERT2 mice (the Jackson Laboratory, Strain #:025124). To obtain abdominal adipose tissue‐specific Chchd10 overexpressing (ATC10‐OE) mice and their relative control (Ctrl) mice, seven‐week‐old male wildtype mice with C57BL/6N background were subjected to intraperitoneal injection^[^ ^78^ ^]^ of customized adeno‐associated virus (AAV) of Rec 2 serotype expressing luciferase as control (Rec2Ctrl) or Chchd10 (Rec2C10) with the dosage of 2*10^10^ vg per mice^[^ ^78^ ^]^ for once. All mice were housed under temperature‐controlled conditions (23 °C, 12‐hour light/dark cycle, 60–70% humidity) in NKP mouse cages with woodchip bedding and unrestricted access to food and drink. Mice were allocated to their experimental groups according to their genotypes. No randomization of mice was used. The investigators were not blinded to the experimental groups. All mouse experiments followed the ARRIVE guidelines (http://www.nc3rs.org.uk/ARRIVEpdf). All experimental protocols were approved by the Committee on the Use of Live Animals in Teaching and Research at the University of Hong Kong (number: 4784‐18).

To induce short‐term excess energy intake and chronic diet‐induced obesity (DIO) models, six‐week‐old male C57BL/6N mice were subjected to a high‐fat diet (HFD, 45% calorie from fat, Research Diet # D12451) for 3 days and 10 weeks, respectively (*n* = 5). To determine the change of Chchd10 expression in adipose tissue of mice subjected to repeated STC and HFD feeding, six‐week‐old male C57BL/6N mice fed with STC were subjected to HFD feeding for 3 days and then changed to STC feeding for another 3 days (*n* = 5). To determine the effect of Chchd10 deficiency in short‐term excess energy intake and DIO models, six‐week‐old male ATC10‐KO mice and C10‐WT mice were subjected to HFD or STC feeding for 3 days (*n* = 6) and 10 weeks (*n* = 12), respectively. To determine the effect of Chchd10 overexpression in DIO models, eight‐week‐old ATC10‐OE mice and Ctrl mice, which received AAV injection for 1 week, were subjected to HFD or STC feeding for 4 weeks (*n* = 8). To determine the effect of extensive knockout of Chchd10 in adipose tissue during HFD feeding, 10‐week‐old Chchd10^f/f^ mice and Chchd10^f/f+Adq‐iCreERT2^ mice fed with HFD for 4 weeks were subjected to intraperitoneal injection of Tamoxifen (75 mg kg^−1^ body weight) for five consecutive days. Afterward, mice were subjected to HFD feeding for an additional 4 weeks (*n* = 6). The mouse body weight was recorded weekly, and the body composition (fat, lean, and fluid) was examined by The Minispec LF90 Body Composition Analyzer (Bruker) weekly or biweekly. Mice were sacrificed at the endpoint without starvation.

### Glucose and Insulin Tolerance Test

The mouse glucose and insulin tolerance tests were conducted according to the guidelines for metabolic tolerance tests in mice, as previously reported.^[^ ^79^ ^]^ For the Glucose tolerance test, six‐week‐old male ATC10‐KO mice and C10‐WT mice fed with HFD or STC for 8 weeks were housed in clean cages with food deprivation for 6 h before intraperitoneal injection of D‐glucose (1.5 g kg^−1^ lean mass). Blood glucose was examined at 0, 15, 30, 45, 60, 90, and 120 min after injection. For the insulin tolerance test, six‐week‐old male ATC10‐KO mice and C10‐WT mice fed with HFD or STC for 9 weeks were fasted for 6 h, followed by intraperitoneal insulin injection (1.5 U kg^−1^ lean mass). Blood glucose was examined at 0, 15, 30, 45, 60, 90, and 120 min after insulin injection. The tolerance of glucose and insulin was indicated by the calculation of the area under the curve.

### Indirect Calorimetry

The whole‐body oxygen consumption was assessed using indirect calorimetry with the Comprehensive Lab Animal Monitoring System (CLAMS, Columbus Instruments), as previously described.^[^ ^80^ ^]^ Briefly, 6‐week‐old male ATC10‐KO mice and C10‐WT mice were subjected to STC or HFD feeding for 8 weeks. Mice were then acclimated to CLAMS cages individually with food and water ad libitum for 24 h. Data of OCR was recorded every 10 or 11 mins during a 48‐hour period at 23 °C. For calculation of energy expenditure: Energy Expenditure: CV = 3.815 + 1.232* RER Heat (Kcal h^−1^) = CV × VO_2_. Where: CV is the calorific value (the relationship between heat and the volume of oxygen consumption).^[^ ^81^ ^]^ The calculated energy expenditure was then normalized with the fat or lean mass of mice.

### Stromal Vascular Fraction Isolation and White Adipocyte Differentiation

The SVF was isolated from iWAT of 6‐week‐old male ATC10‐KO or C10‐WT mice by digestion with 0.15% collagenase I and followed with filtration through 70 µm cell strainer and centrifugation. The primary cells were differentiation into white adipocytes by treating cells with induction medium of 10% fetal bovine serum (FBS) and 1% antibiotics penicillin/streptomycin/amphotericin B (PSF) supplemented high glucose DMEM containing dexamethasone (1µM, D4902, Sigma‐Aldrich), isobutylmethylxanthine (0.5 mm, I5879, Sigma‐Aldrich), rosiglitazone (1µM, PHR2932, Sigma‐Aldrich), and recombinant human insulin (10µg mL^−1^, #11 376 497 001, Roche) for two days. The induced cells were then followed with the replacement of a maintenance medium containing insulin (10µg mL^−1^) every two days.

### Cell Culture

To examine the dynamic change of Chchd10 in response to glucose and fatty acid stimulations, 4‐day‐differentiated WT adipocytes were treated with glucose or oleic acid at various concentrations for 6 h. To establish the in vitro insulin‐resistant (IR) model, 4‐day‐differentiated Chchd10 KO adipocytes and WT adipocytes were subjected to the treatment described previously with slight modifications.^[^ ^42^ ^]^ Briefly, IR was induced with the treatment of both TNFα (2.5 nm, 410‐MT, R&D System) and hypoxia (1% oxygen) for 16 h. To knock down GSTA4 expression, 2‐day‐differentiated Chchd10 KO and WT adipocytes were infected with adenovirus encoding GSTA4 shRNA (Ad‐shGSTA4) or scramble RNA (Ad‐scramble) for 48 h. To inhibit NRF2, p62, or mTORC1, 2‐day‐differentiated adipocytes were treated with ML385 (10µm, HY‐100523, MedChemExpress), K67 (10µm, HY‐111126, MedChemExpress), or rapamycin (200 nM, 553 210, Sigma‐Aldrich) for 48 h, respectively. To knock down NRF2 and TDP43 expression, 2‐day‐differentiated adipocytes were transfected with mouse NFE2L2 siRNA (siNRF2) targeting 3′ UTR (mm.Ri.Nfe2l2.13.1, Integrated DNA Technologies), mouse Tardbp siRNA (siTDP43) targeting exon 3 and 4 (mm.Ri.Tardbp.13.2, Integrated DNA Technologies), or scramble siRNA as control (siCTR, #51‐01‐14, Integrated DNA Technologies) using lipofectamine 3000 transfection system (Invitrogen) for 48 h. To assess Raptor RNA stability, 4‐day‐differentiated adipocytes were treated with actinomycin D (1µg mL^−1^, A9415, Sigma‐Aldrich) or PBS for 0, 2, or 4 h. The RNA was then extracted, reverse transcribed into cDNA, and subjected to qPCR analysis. To obtain Chchd10 overexpressing white adipocytes, 3T3L1 cells were transfected with lentivirus‐encoded mouse CHCHD10 cDNA. The stable Chchd10 overexpressing (C10 OE) and relative control (Ctrl) cells were subjected to white adipocyte differentiation, as mentioned above.

### Western Blot Analysis

The total protein was extracted from adipocytes or adipose tissues with radioimmunoprecipitation assay (RIPA) buffer by sonication or tissue homogenization, respectively. The crude protein lysates were kept on ice for 30 min and followed by centrifugation at 14 000 rpm at 4 °C for 15 min. The nuclear and cytoplasmic protein lysate was extracted from adipocyte using NE‐PER nuclear and cytoplasmic extraction reagents (#78 835, Thermo Scientific) according to the manufacturer's instructions. The protein concentration was measured by Pierce bicinchoninic acid (BCA) protein assay kit (#23 225, Thermo Scientific). Protein samples were denatured and separated by electrophoresis using SDS‐polyacrylamide gel and transferred to polyvinylidene difluoride (PVDF) membranes. The membranes were blocked with TBST containing 10% bovine serum albumin (BSA) and probed with primary antibodies against Chchd10 (25671‐1‐AP) and GAPDH (60004‐1‐Ig) from Proteintech; HSP90 (#4874), PPARγ (#2443), CEBPα (#2295), p62 (#5114), Keap1 (#8047), Lamin B1 (#13 435), and p‐p62 (S349, #16 177) from Cell Signaling Technology; NRF2 (ab31163) and nucleophosmin 1 (NPM1, ab15440) from Abcam, FAS (sc‐48357), Raptor (sc‐81537), and TDP43 (sc‐376311) from Santa Cruz; GSTA4 (#PA5‐106329) from Invitrogen, and p‐p62 (S351, MBS9212103) from MyBioSource at dilutions recommended by the suppliers. After overnight incubation at 4 °C, the membranes were washed with TBST and probed with respective peroxidase‐conjugated secondary antibodies (Cell Signaling Technology) for one hour at room temperature. The protein bands were developed with clarity max ECL substrate (#1 705 062, Vazyme). The expression of the target protein was normalized against the cytoplasmic housekeeping protein HSP90 or GAPDH or the nuclear housekeeping protein Lamin B1 or NPM1.

### Histological Analysis of Adipose Tissue

To assess the morphologic change of adipose tissue, hematoxylin and eosin (H and E) staining was performed. Mouse adipose tissue specimens were fixed in 4% (w v^−1^) formaldehyde in PBS, then dehydrated and embedded in paraffin as previously described.^[^ ^82^ ^]^ The paraffin‐embedded tissue sections (5µm) were deparaffinized, rehydrated, and stained with hematoxylin (H3136, Sigma‐Aldrich) and eosin (HT110132, Sigma‐Aldrich) solutions according to the manufacturer's instructions. Slides were imaged using a conventional brightfield microscope with at least six low‐power fields in a blinded fashion. The size of adipocytes was quantified using Image‐J software (NIH, USA) with Adiposoft plugin.

### Oil Red O Staining for Mouse Liver and Adipocytes

To access the neutral lipid accumulation in mouse liver and adipocytes in culture, the frozen liver sections (8µm) and coverslips with adipocytes were fixed with 4% (w v^−1^) formaldehyde in PBS for 10 mins and followed by PBS wash 3 times. The samples (sections and cell coverslips) were then rinsed with 60% isopropanol, stained with 0.3% Oil Red O (O0625, Sigma‐Aldrich) solution for 15 min, and followed with another rinse of 60% isopropanol. The sections were quickly stained with hematoxylin to visualize nuclei. Afterward, samples were washed with ddH2O and mounted with an aqueous mounting medium. Slides and coverslips were imaged using a conventional brightfield microscope with at least six low‐power fields in a blinded fashion. The accumulation of lipids in the liver was quantified by the Oil Red O positive area using Image‐J software (NIH, USA). The accumulation of lipids in the adipocytes was quantified by the optical density of Oil Red O dissolved from adipocytes by absolute isopropanol at 515 nm wavelength.

### Hepatic Triglyceride Measurement

To extract lipids from mouse liver, 50 mg liver samples were homogenized with 200 µL PBS. The tissue lysates were then mixed with 5 mL chloroform/methanol (2:1, v/v, with 151µM butylated hydroxytoluene) on rotor overnight at 4 °C. Afterward, 1 mL mixture was added with 200 µL saline and centrifuged at 5000 rcf for 15 min at room temperature. After centrifugation, 200 µL samples from the lower chloroform layer were collected and subjected to air drying. The lipid extracts were then dissolved with 100 µL absolute ethanol and subjected to triglyceride measurement using a Triglyceride Liquicolor assay kit (#2199‐430, StanBio).

### Measurement of Oxygen Consumption Rate

To measure the basal OCR of adipose tissues, freshly isolated mouse iWAT, eWAT, and BAT (≈3 mg) were rinsed in PBS and concealed in XF24 islet capture microplate (#101122‐100, Agilent Technologies) with incubation of Agilent seahorse XF assay medium (#103680‐100, Agilent Technologies). The samples were then analyzed using the Agilent Seahorse XF24 analyzer (Agilent Technologies). To assess the mitochondrial respiratory function of Chchd10 KO and WT adipocytes subjected to the in vitro IR model or normal culture condition, cells were washed once with PBS and incubated in 500 µL XF‐DMEM. After 1 hour, cells were subjected to an Agilent Seahorse XF24 analyzer with series injections of oligomycin (5µm), FCCP (0.3µm), and rotenone (2µm) plus antimycin A (2µm). The basal respiration, ATP‐linked respiration, proton leak, maximal respiration capacity, reserved capacity, and non‐mitochondrial respiration were analyzed.

### ATP Measurement

ATP was extracted from mouse iWAT and eWAT, referring to previous instructions.^[^ ^83^ ^]^ Briefly, 200 mg tissues were homogenized with ice‐cold phenol‐TE. 1 mL tissue lysate was added with 200 µL Chloroform and 150 µL deionized water and subjected to centrifugation at 10 000 rcf for 5 mins. The upper aqueous phase was collected and diluted with deionized water. The ATP abundance in diluted extracts was then measured with an ATP Determination Kit (A22066, Invitrogen) according to the manufacturer's instructions. The ATP levels indicated by luminescence were then normalized by the tissue weight.

### Transmission Electron Microscopy

Freshly isolated mouse iWAT, eWAT, and BAT (≈2 mm cubes) were fixed in 2.5% glutaraldehyde in cacodylate buffer (0.1 m sodium cacodylate‐HCl buffer pH 7.4) for 16 h at 4 °C. Samples were then transferred to cacodylate buffer with 0.1 m sucrose to stop fixation and send to Electron Microscope Unit of the University of Hong Kong for further processing, sectioning, and staining for transmission electron microscopy. The samples were then imaged using a Philips CM100 transmission electron microscope (Philips) attached to an Olympus SIS Tengra CCD Camera (2.3k × 2.3k pixels). Five animals per group were examined, and ten electron micrographs of the mitochondrial area of each adipose depot were captured.

### Quantitative PCR (qPCR)

Total RNA was extracted from adipocytes or tissues with RNAiso Plus (9109, Takara Bio Inc) according to the manufacturer's instructions. Complementary DNA (cDNA) was prepared using a Primescript RT‐PCR kit (RR037A, Takara Bio Inc). Quantitative PCR was performed using SYBR Premix Ex Taq (RR420A, Takara Bio Inc) and the 7900 HT PCR machine (Applied Biosystems). The relative mRNA expression of a specific gene was measured with the 2−∆∆Ct method and normalized against the housekeeping gene *GAPDH*. All the primers were purchased from Integrated DNA Technologies. The sequences are listed in Table S1 of Supporting Information.

### Biochemical Assays

The abundance of 4‐HNE protein adducts in mouse eWAT was measured with lipid peroxidation (4‐HNE) assay kit (ab238538, Abcam) according to the manufacturer's instruction. The abundance of carbonylated protein in mouse eWAT was measured with Protein Carbonyl Content Assay Kit (ab126287, Abcam) according to the manufacturer's instructions.

### RNA Sequencing and Analysis

RNA‐seq experiments were performed using RNA extracts of iWAT of 5 animals per group. The RNA samples (total RNA ≥ 200 ng, concentration ≥ 20 ng µL^−1^. RIN ≥ 7.0, 28S/18S ≥ 1.0) extracted by RNAiso Plus (9109, Takara Bio Inc) and purified by Quick‐RNA Miniprep Kit (R1054, Zymo Research) were subjected to the DNBSEQ Eukaryotic Strand resequencing and analysis service provided by Beijing Genomics Institute (BGI). Reads of each sample were aligned to the mouse genome (TXID 10 090, GRCm38.p6). Differentially expressed genes were those that had adjusted *p‐*value < 0.05. The TPM value of samples was subjected to the gene set enrichment analysis (GSEA). The enriched pathways with adjusted *p‐*value < 0.05 were recognized as significantly altered signaling pathways.

### Gene Expression Analysis Based on the GEO Dataset

The GEO dataset (GSE65557) was used to analyze the gene expression of Chchd10. It is a microarray data of adipocytes and stroma vascular cells (SVC) in VAT from 8‐week‐old male C57BL/6J mice treated with 60% HFD for 0 days (STC), 3 days, and 7 days.^[^ ^20^ ^]^ The volcano plot was generated based on the log *p*‐value and log fold change by GEO2R. The expression value of Chchd10 was compared among adipocytes in VAT of mice fed with HFD for 0, 3, and 7 days and between the adipocytes and SVC in VAT of mice fed with STC.

### Statistical Analysis

All the replicate experiments, including in vivo and in vitro experiments, were repeated at least two times. All statistical analyses were performed using Prism 10 software (GraphPad). No data was excluded or transformed in the statistical analysis. All data was expressed as mean ± SD. The animal sample size for each study was selected based on literature documenting similar well‐characterized experiments.^[^ ^84^ ^]^ Data normality was accessed using the Shapiro‐Wilk test. Differences between the two groups were evaluated using the Student's *t*‐test for normal distributions or the Mann‐Whitney *U* test for non‐normal distributions. Differences among multiple groups were compared using ANOVA, followed by Turkey's or Šídák multiple comparison tests. All statistical tests were two‐tailed. *p* values less than 0.05 were considered to indicate statistically significant differences.

## Conflict of Interest

The authors declare no conflict of interest.

## Author Contributions

X.W and Z.Z contributed equally to this work. R.L.C.H. and X.W. designed the study, and X.W., Z.Z., J.L., J.Z., L.Y., L.S., L.Y.C., X.H., M.J., and Z.P. conducted the experiments and analyzed the data; X.W. and Z.Z. prepared the figures and drafted the manuscript; R.L.C.H. edited the manuscript; A.X. and R.L.C.H. approved the manuscript.

## Supporting information



Supporting Information

## Data Availability

The data that support the findings of this study are available from the corresponding author upon reasonable request.
