# Supplementary material for: Chchd10: A Novel Metabolic Sensor Modulating Adipose Tissue Homeostasis
Source: Adv Sci (Weinh). 2025 Feb 22;12(15):2408763. doi: 10.1002/advs.202408763 (PMC12005791; doi:10.1002/advs.202408763)
Supplement: Supplementary file 1 — Supporting Information [file ADVS-12-2408763-s001.pdf]

ADVANCED SCIENCE
Open Access

Supporting Information

for *Adv. Sci.*, DOI 10.1002/advs.202408763

Chchd10: A Novel Metabolic Sensor Modulating Adipose Tissue Homeostasis

*Xiaoping Wu, Zixuan Zhang, Jingjing Li, Jiuyu Zong, Lufengzi Yuan, Lingling Shu, Lai Yee Cheong, Xiaowen Huang, Mengxue Jiang, Zhihui Ping, Aimin Xu and Ruby L.C. Hoo**

Fig.S1

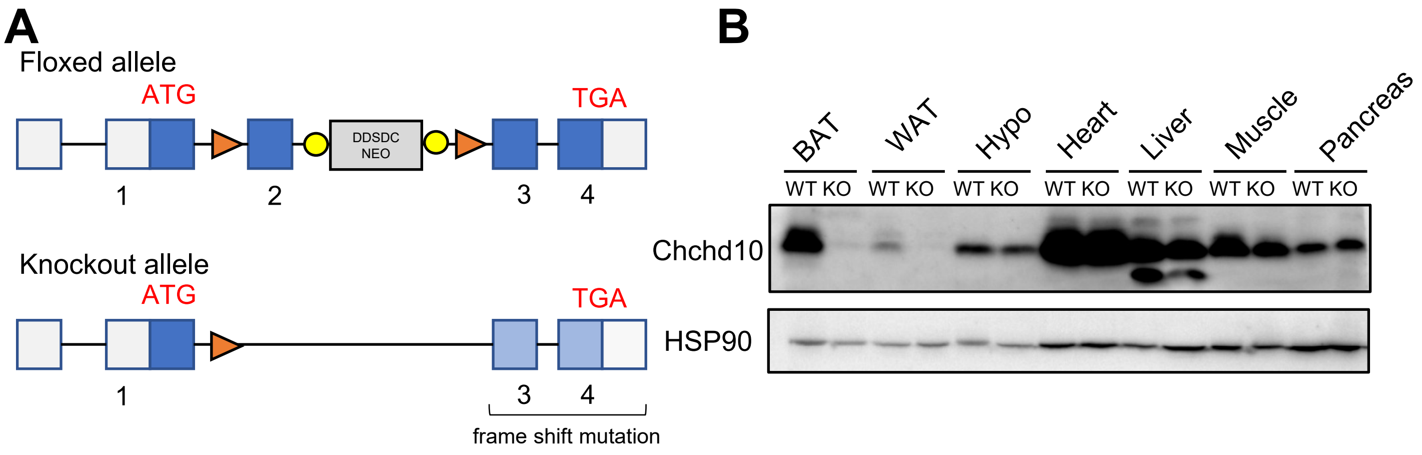

Fig.S1 | Establishment of adipose tissue-specific Chchd10 knockout mice

(A) Schematic diagram showing CHCHD10 floxed and knockout alleles. (Lines = introns; closed boxes = exons; blue areas = coding regions; arrowheads = loxP sites).

(B) Representative immunoblots of the protein abundance of Chchd10 and HSP90 in various tissues and organs of C10-WT and ATC10-KO mice.

Fig.S2

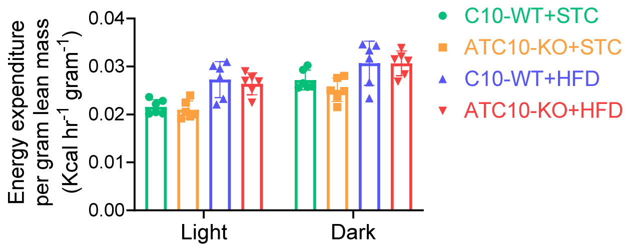

Fig.S2 | Chchd10 deficiency did not alter lean mass-associated energy expenditure in mice.

Calculated energy expenditure of Fig.3B normalized with lean mass weight (n=6). Data are presented as mean ± SD and statistical significance was analyzed by two-way ANOVA.

Fig.S3

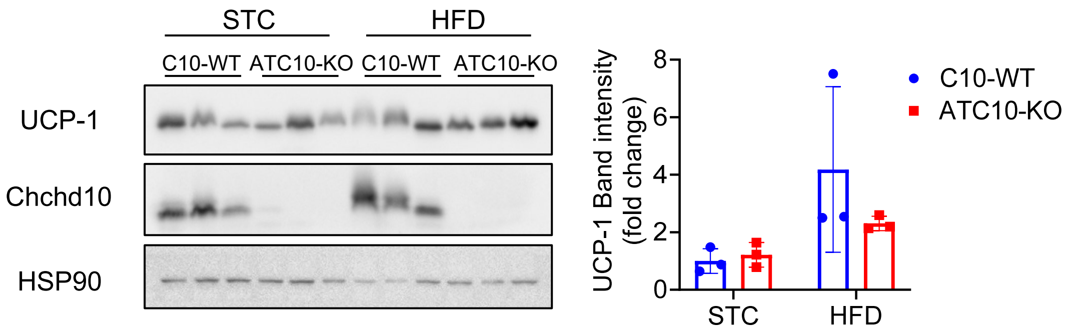

Fig.S3 | Chchd10 deficiency did not alter the expression of UCP-1 in BAT.

Representative immunoblots of the protein abundance of UCP-1 and GAPDH in BAT of mice subjected to long-term feeding model. The right panel is the quantification of UCP-1 band intensity normalized with HSP90 (n = 3). Data are presented as mean ± SD. Statistical significance was analyzed by two-way ANOVA.

Fig.S4

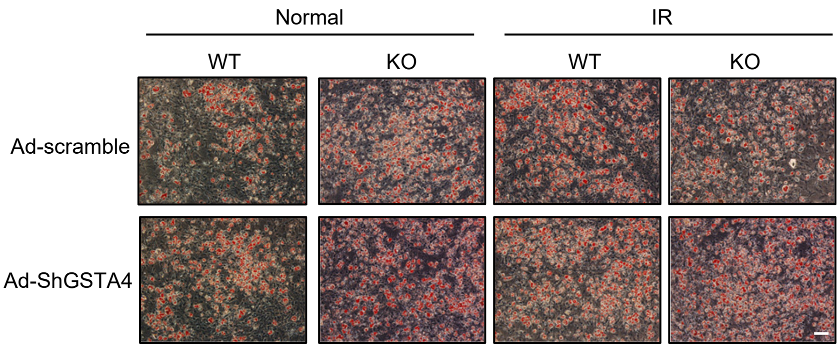

Fig.S4 | Chchd10 deficiency attenuates lipid accumulation under insulin-resistant conditions in a GSTA4-dependent manner.

Representative images of Oil Red O staining of adipocytes indicated in Fig.6L (scale bar = 100μm).

Fig.S5

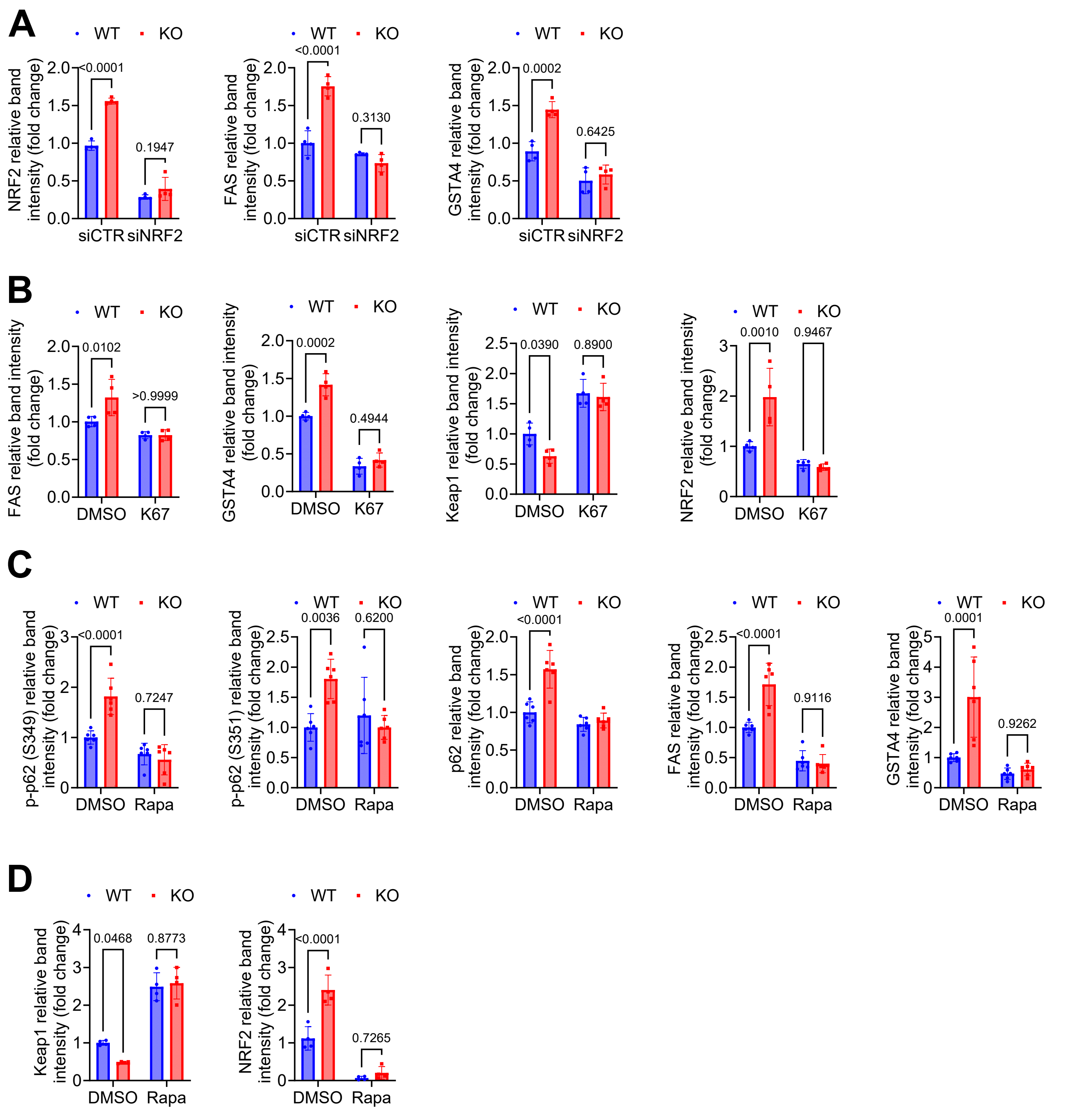

Fig.S5 | Chchd10 deficiency promotes adipogenesis and GSTA4 expression by activating p62/Keap1/NRF2 axis

The quantifications of western blot results in (A) Fig.7D (n = 4), (B) Fig.7F (n=4), (C) Fig.7G (n= 4 ~ 6), and (D) Fig.7H (n = 4).

Data are presented as mean ± SD. Statistical significance was analyzed by two-way ANOVA (A, B, C, and D).

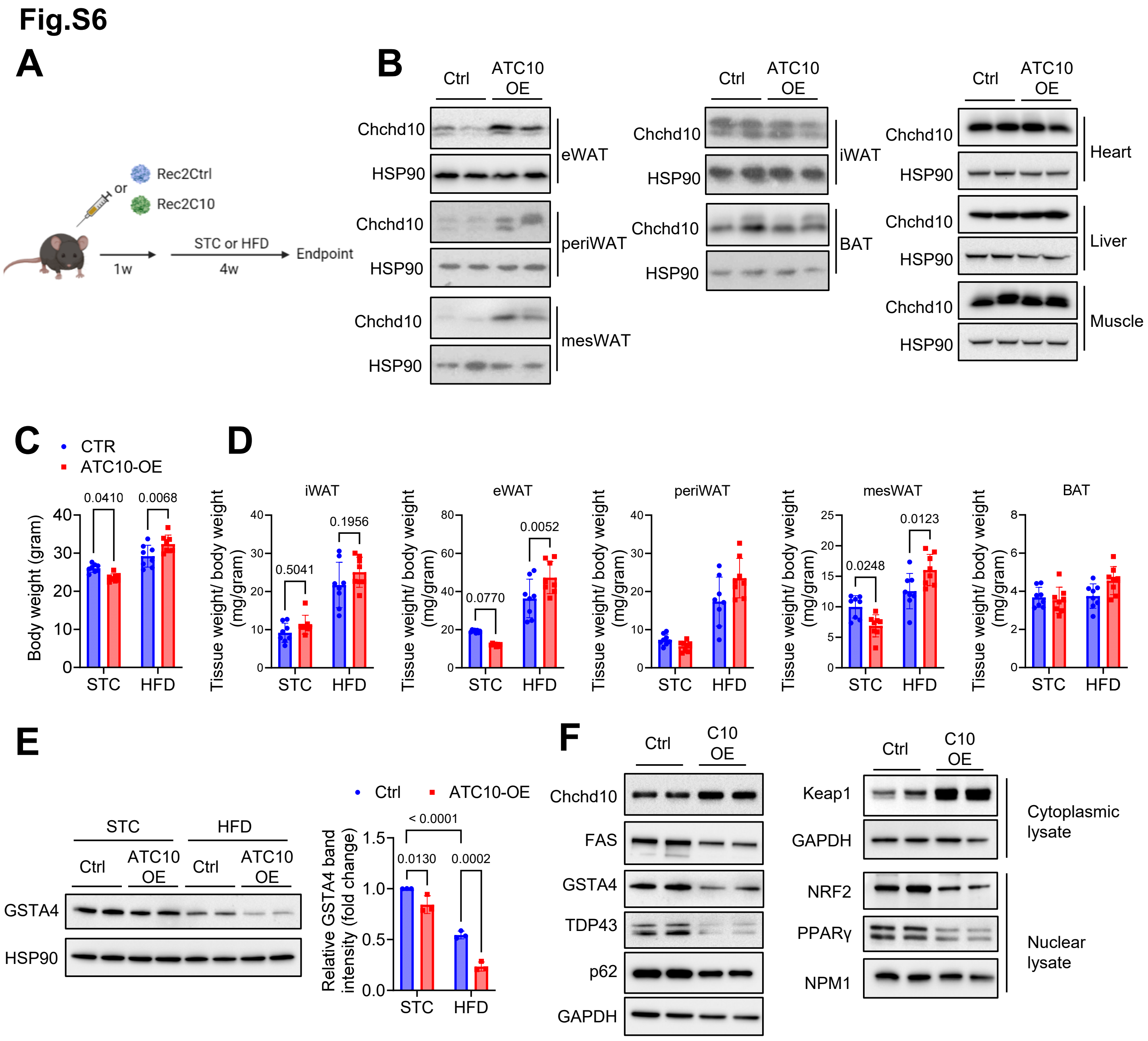

Fig.S6 | Abdominal adipose tissue-specific Chchd10 overexpression reduces adiposity in STC-fed mice while exaggerates obesity in HFD-fed mice

Six-week-old male C57BL/6N mice were subjected to intraperitoneal injection of adeno-associated virus of Rec2 serotype expressing luciferase control (Rec2Ctrl) or Chchd10 (Rec2C10) for 1 week and subsequently subjected to STC or HFD feeding for 4 weeks (n=8).

(A) Schematic diagram showing abdominal specific Chchd10 overexpression and the subsequent diet induction.

(B) Representative immunoblots of the protein abundance of Chchd10 and HSP90 in abdominal adipose tissues, peripheral adipose tissues, and major organs/tissues of mice.

(C) Mouse body weight (n = 8).

(D) Mouse tissue weight normalized with body weight (n = 8).

(E) Representative immunoblots of the protein abundance of GSTA4 and HSP90 in mouse eWAT. The right panel is the quantification of GSTA4 band intensity normalized with HSP90 (n = 3).

(F) Representative immunoblots of the protein abundance of Chchd10, FAS, GSTA4, and GAPDH in the whole cell lysate, Keap1 and GAPDH in the cytoplasmic lysate, and NRF2, PPARγ, and NPM1 in the nuclear lysate of control (Ctrl) and Chchd10 overexpressing (C10 OE) 3T3L1 cells subjected to 4-day differentiation of white adipocytes.

Data are presented as mean ± SD. Statistical significance was analyzed by two-way ANOVA (C, D, and E).

Fig.S7

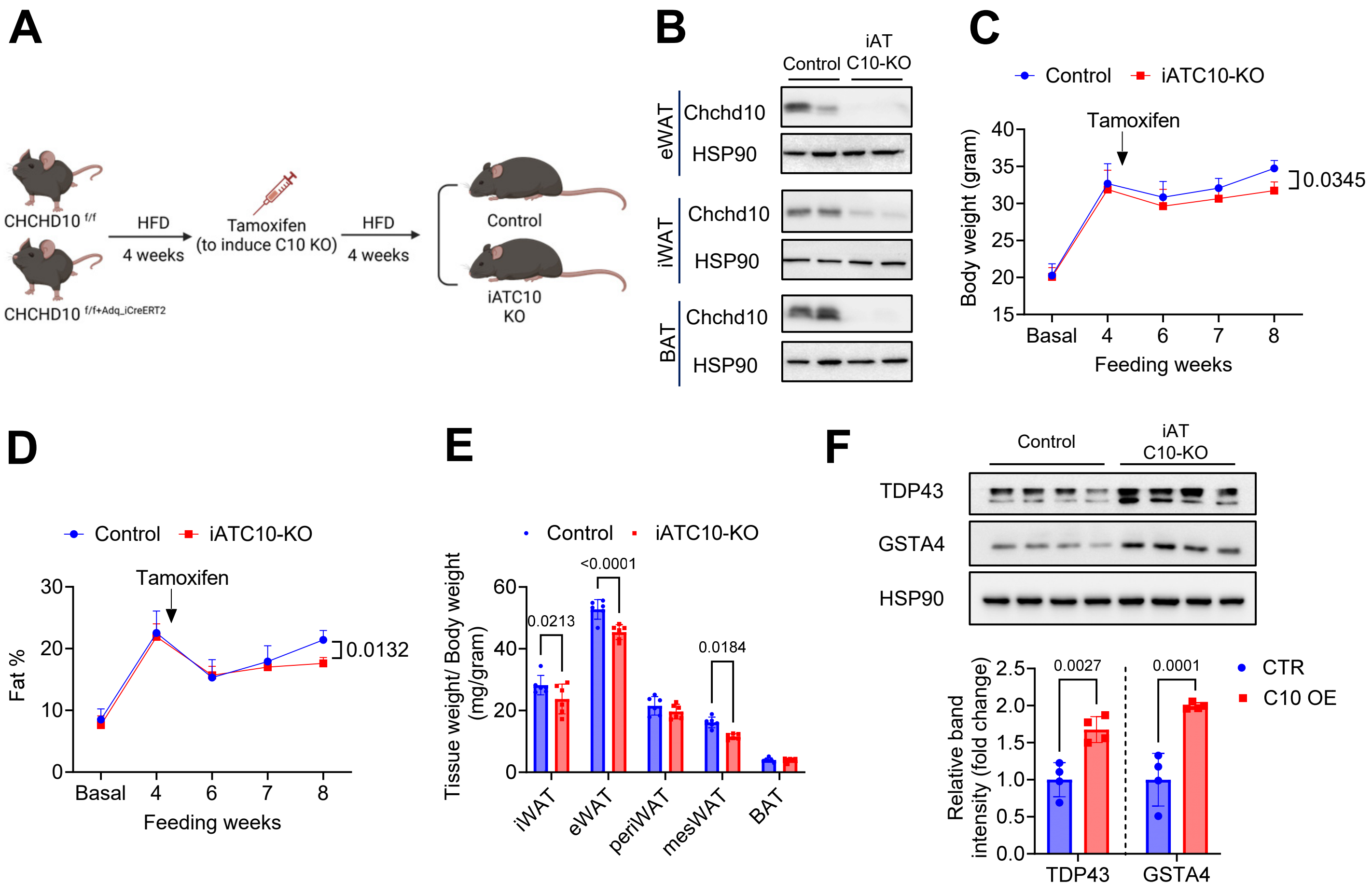

Fig.S7 | Tamoxifen-induced conditional adipose tissue-specific Chchd10 knockout decelerates the progression of diet-induced obesity in mice

Six-week-old male CHCHD10$^{f/f}$ mice and CHCHD10$^{f/f+Adq_iCreERT}$ mice were subjected to HFD feeding for 8 weeks. In the middle of the feeding period, mice were injected with tamoxifen for 5 consecutive days to induce conditional Chchd10 knockout (n = 6).

(A) Schematic diagram of establishing inducible adipose tissue-specific Chchd10 knockout (iATC10-KO) and relative control mice.

(B) Representative immunoblots of the protein abundance of Chchd10 and HSP90 in iWAT, eWAT, and BAT.

(C) Dynamic change of mouse body weight (n = 6).

(D) Dynamic change of mouse fat mass percentage (n = 6).

(E) Mouse fat pad weight normalized with body weight (n = 6).

(F) Representative immunoblots of the protein abundance of TDP43, GSTA4, and HSP90 in mouse eWAT. The lower panel is the quantification of TDP43 and GSTA4 band intensity normalized with HSP90 (n = 4).

Data are presented as mean ± SD. Statistical significance was analyzed by two-way ANOVA (C, D, E, and F).

Table S1. Sequence of primers used in the present study

F: Forward primer; R: Reverse primer

Name	Sequence (5' to 3')
CEBPβ F	CAACCTGGAGACGCAGCACAAG
CEBPβ R	GCTTGAACAAGTTCCGCAGGGT
CEBPα F	GCAAAGCCAAGAAGTCGGTGGA
CEBPα R	CCTTCTGTTGCGTCTCCACGTT
PPARγ F	GTACTGTCGGTTTCAGAAGTGCC
PPARγ R	ATCTCCGCCAACAGCTTCTCCT
Adiponectin F	AGATGGCACTCCTGGAGAGAAG
Adiponectin R	ACATAAGCGGCTTCTCCAGGCT
F4/80 F	CTTTGGCTATGGGCTTCCAGTC
F4/80 R	GCAAGGAGGACAGAGTTTATCGTG
TNFα F	ACGGCATGGATCTCAAAGAC
TNFα R	AGATAGCAAATCGGCTGACG
*COL1α*1 F	CAATGGTGAGACGTGGAAAC
COL1α1 R	GGTTGGGACAGTCCAGTTCT
COL3α1 F	CTGTAACATGGAAACTGGGGAAA
COL3α1 R	CCATAGCTGAACTGAAAACCACC
GSTA4 F	GATGATTGCCGTGGCTCCATTTA
GSTA4 R	CTGGTTGCCAACGAGAAAAGCC
FAS F	CACAGTGCTCAAAGGACATGCC
FAS R	CACCAGGTGTAGTGCCTTCCTC
Raptor F	CTTCCTATCCGTCTTGGCAGAC
Raptor R	CTCCAGACAGATGGCAATCAGG
GAPDH F	CATCACTGCCACCCAGAAGACTG
GAPDH R	ATGCCAGTGAGCTTCCCGTTCAG

Raw blots of Western blotting

Fig.1

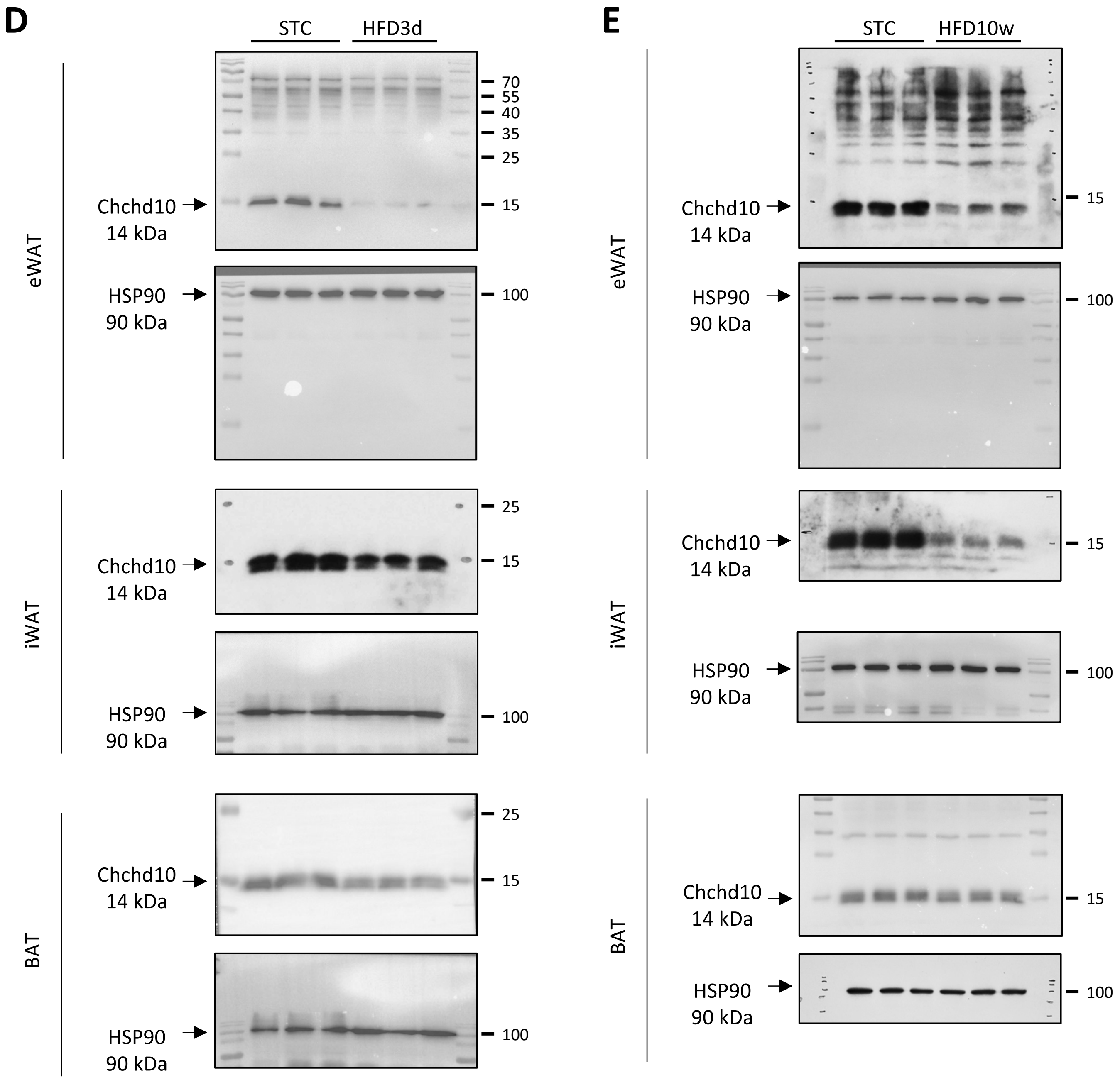

Fig.1

H

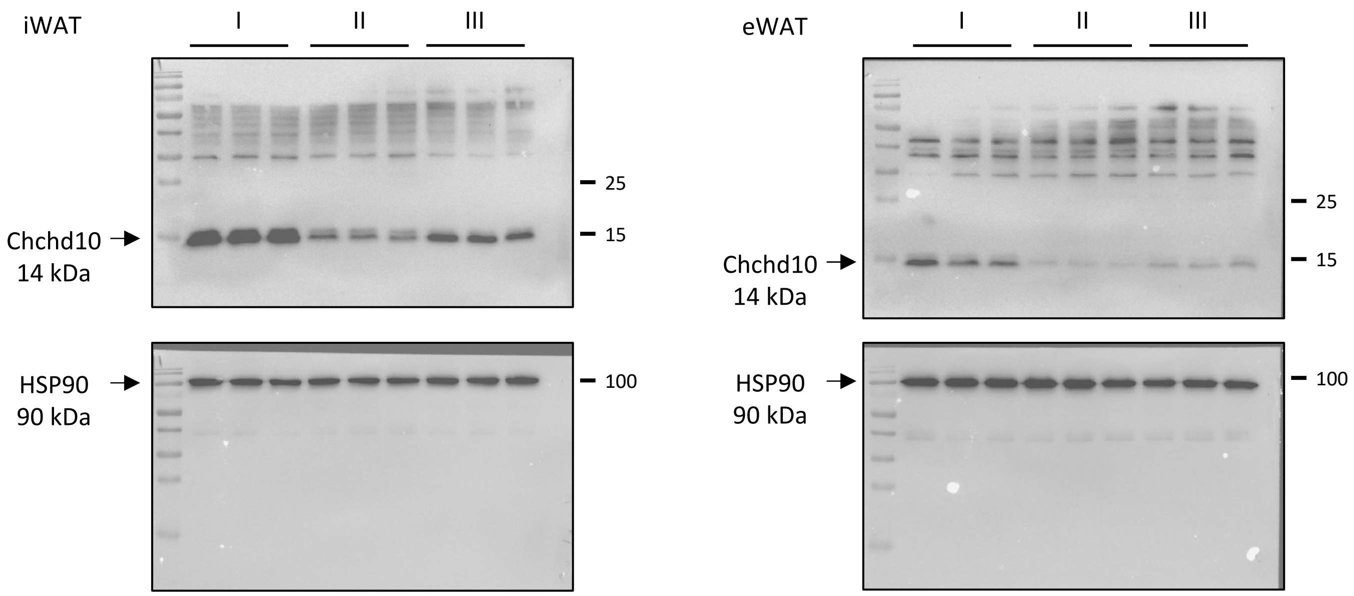
iWAT
I
II
III
25
Chchd10
14 kDa
15
HSP90
90 kDa
100
eWAT
I
II
III
25
Chchd10
14 kDa
15
HSP90
90 kDa
100

Fig.5

G

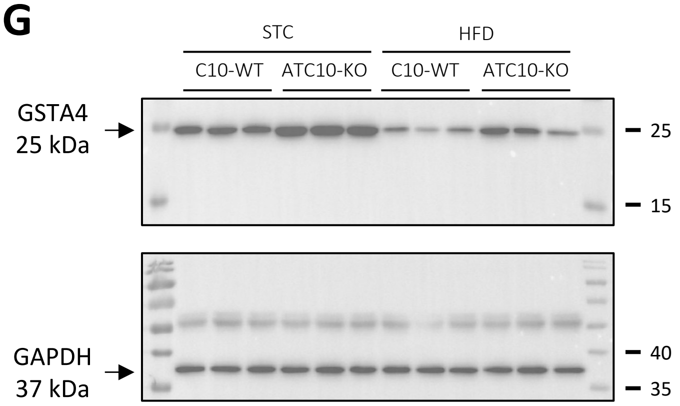
STC
HFD
C10-WT
ATC10-KO
C10-WT
ATC10-KO
GSTA4
25 kDa
25
15
GAPDH
37 kDa
40
35

Fig.6

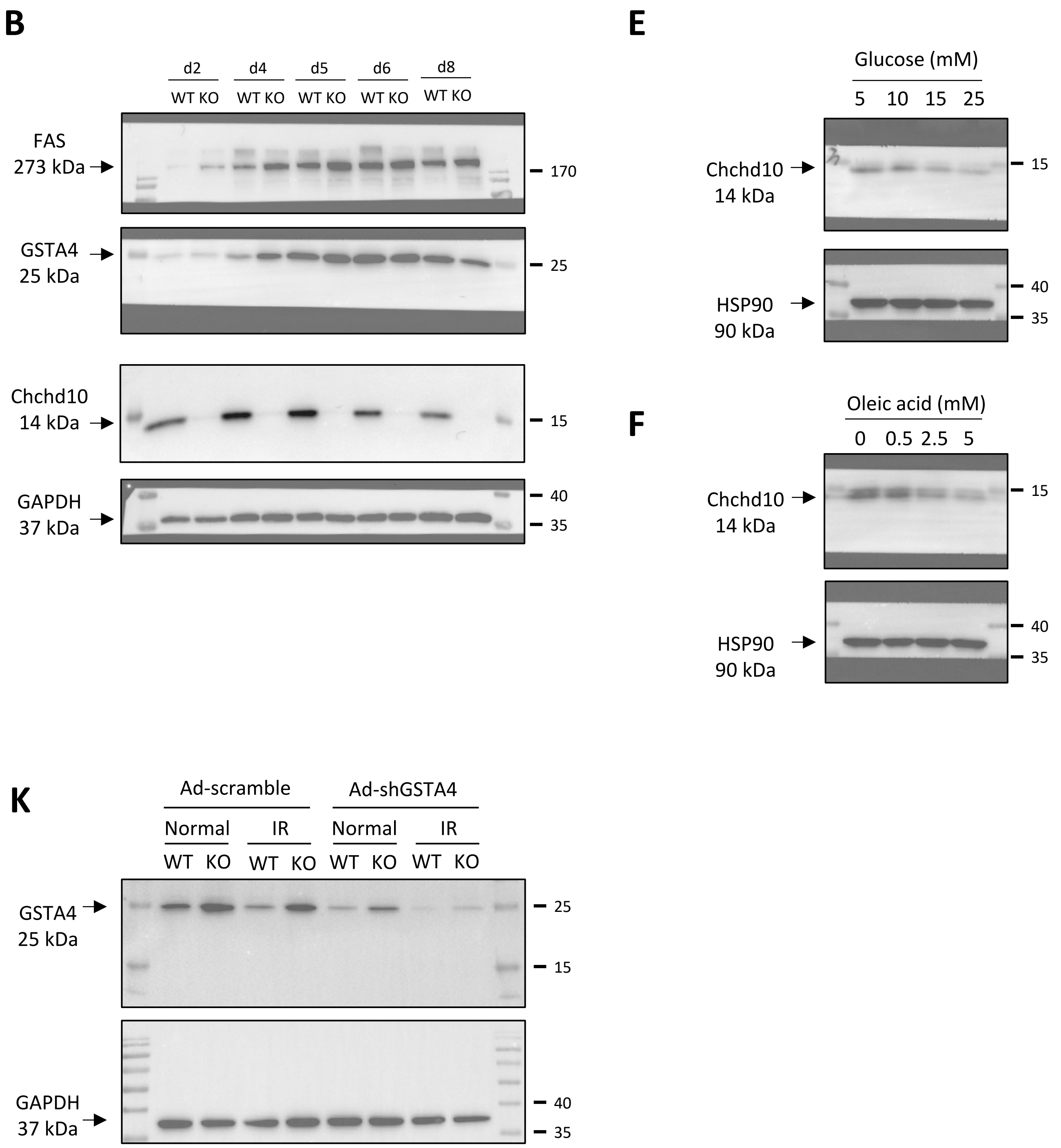

B
d2
d4
d5
d6
d8
WT KO
WT KO
WT KO
WT KO
WT KO
FAS
273 kDa
170
GSTA4
25 kDa
25
Chchd10
14 kDa
15
GAPDH
37 kDa
40
35
E
Glucose (mM)
5 10 15 25
Chchd10
14 kDa
15
HSP90
90 kDa
40
35
F
Oleic acid (mM)
0 0.5 2.5 5
Chchd10
14 kDa
15
HSP90
90 kDa
40
35
K
Ad-scramble
Ad-shGSTA4
Normal
IR
Normal
IR
WT KO
WT KO
WT KO
WT KO
GSTA4
25 kDa
25
15
GAPDH
37 kDa
40
35

Fig.7

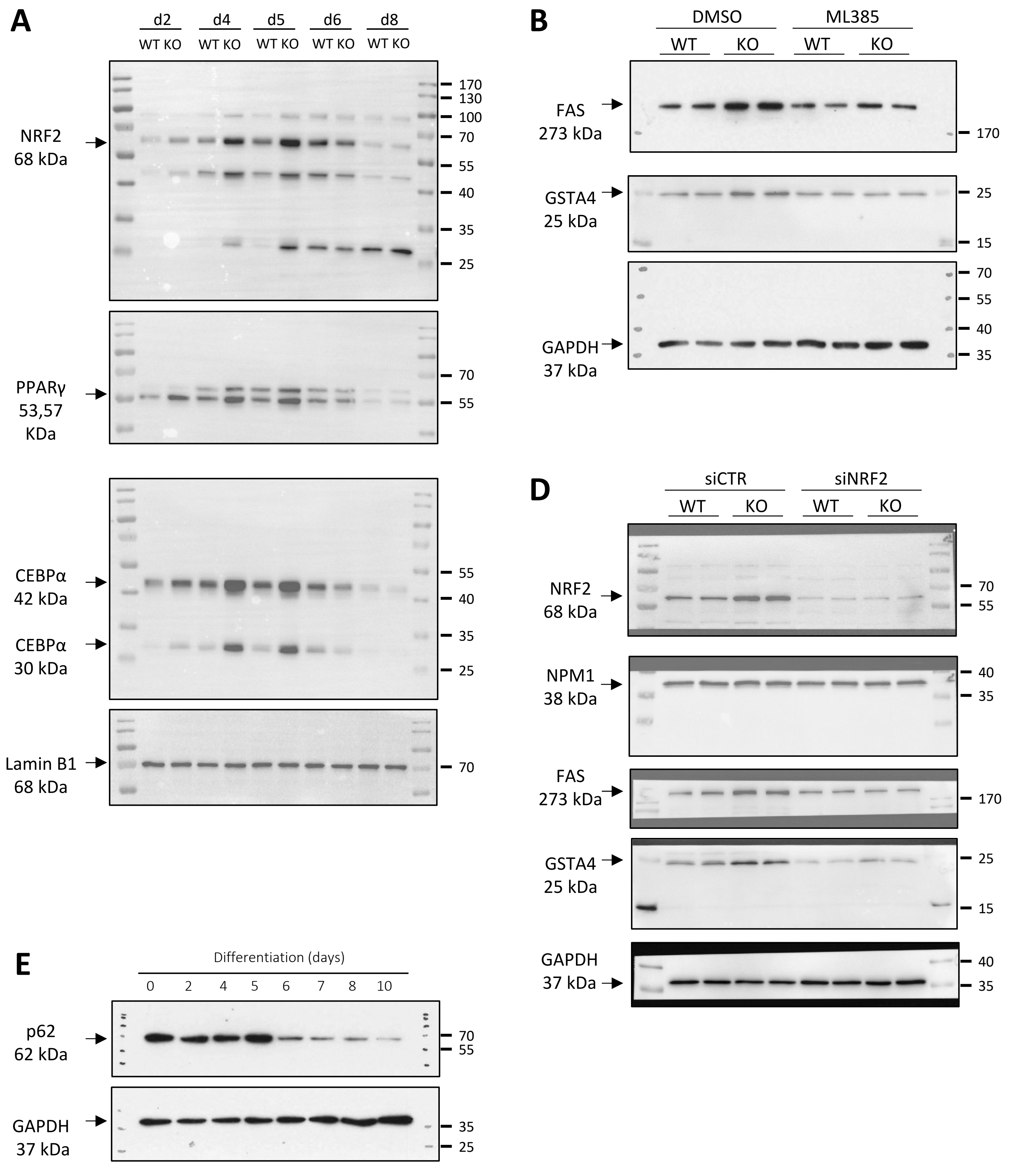
A
d2
d4
d5
d6
d8
WT KO
WT KO
WT KO
WT KO
WT KO
NRF2
68 kDa
170
130
100
70
55
40
35
25
PPARγ
53,57
KDa
70
55
CEBPα
42 kDa
CEBPα
30 kDa
55
40
35
25
Lamin B1
68 kDa
70
B
DMSO
ML385
WT
KO
WT
KO
FAS
273 kDa
170
GSTA4
25 kDa
25
15
GAPDH
37 kDa
70
55
40
35
D
siCTR
siNRF2
WT
KO
WT
KO
NRF2
68 kDa
70
55
NPM1
38 kDa
40
35
FAS
273 kDa
170
GSTA4
25 kDa
25
15
GAPDH
37 kDa
40
35
E
Differentiation (days)
0
2
4
5
6
7
8
10
p62
62 kDa
70
55
GAPDH
37 kDa
35
25

Fig.7

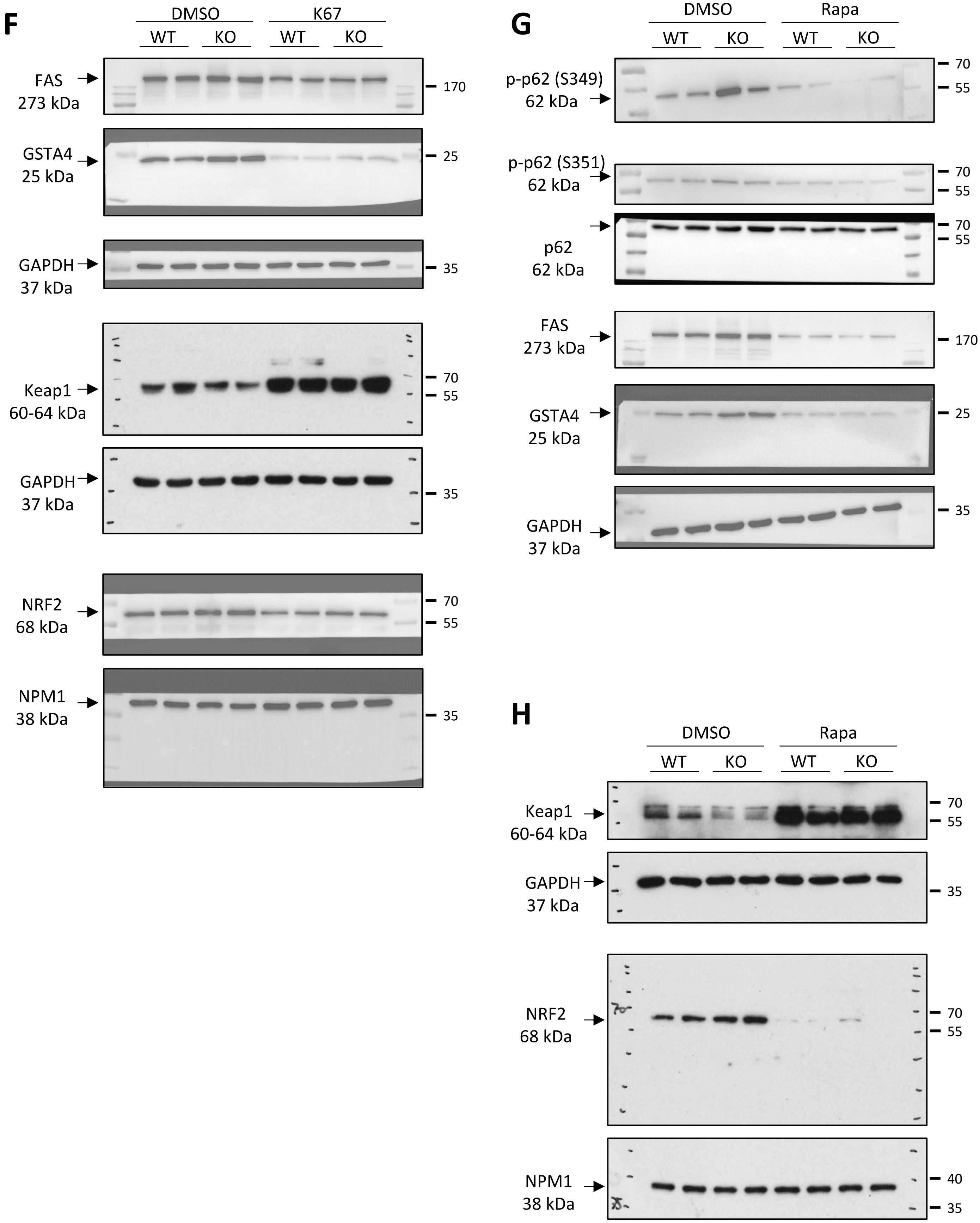
F
DMSO
K67
WT
KO
WT
KO
FAS
273 kDa
170
GSTA4
25 kDa
25
GAPDH
37 kDa
35
Keap1
60-64 kDa
70
55
GAPDH
37 kDa
35
NRF2
68 kDa
70
55
NPM1
38 kDa
35
G
DMSO
Rapa
WT
KO
WT
KO
p-p62 (S349)
62 kDa
70
55
p-p62 (S351)
62 kDa
70
55
p62
62 kDa
70
55
FAS
273 kDa
170
GSTA4
25 kDa
25
GAPDH
37 kDa
35
H
DMSO
Rapa
WT
KO
WT
KO
Keap1
60-64 kDa
70
55
GAPDH
37 kDa
35
NRF2
68 kDa
70
55
NPM1
38 kDa
40
35

Fig.8

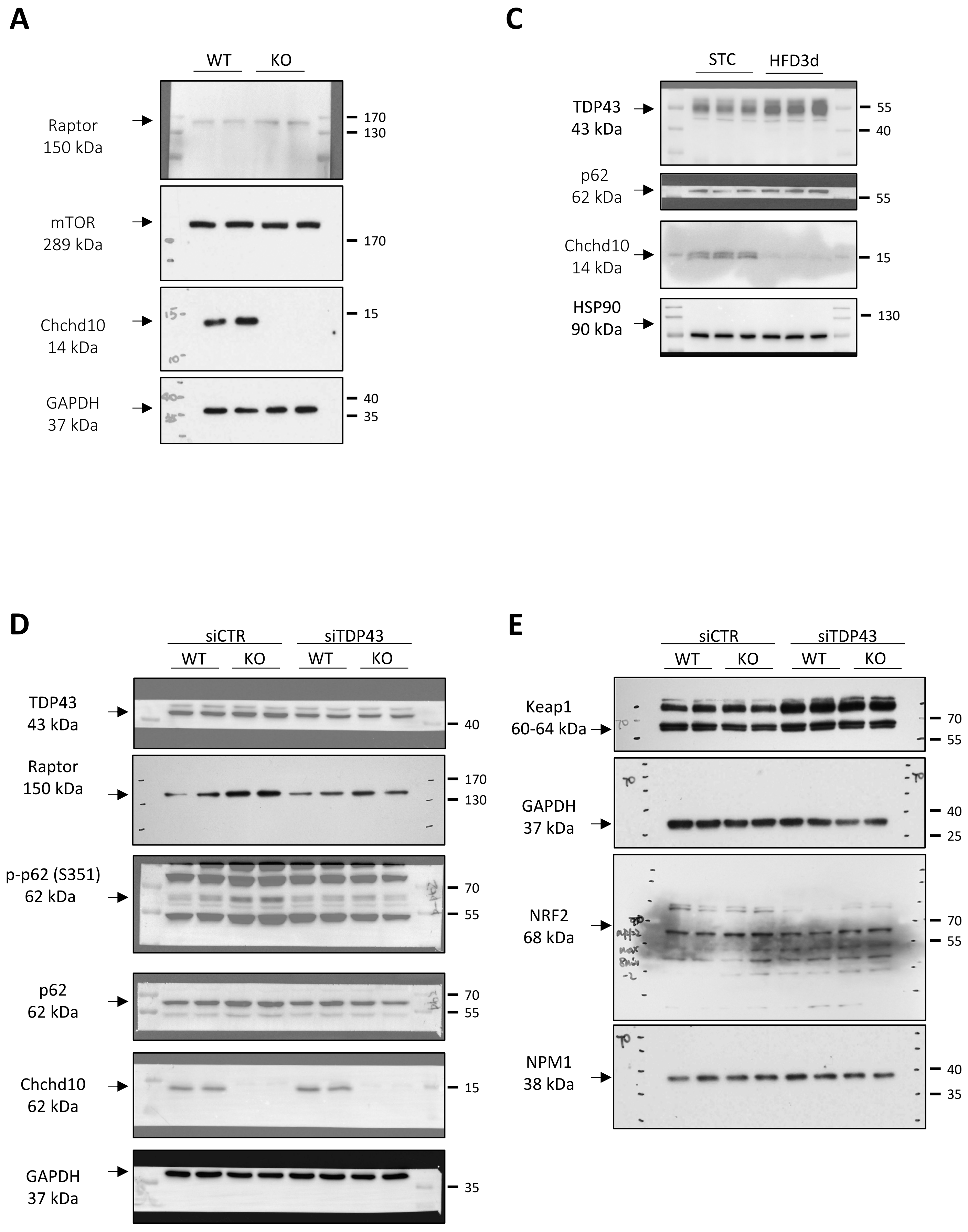
A
WT
KO
Raptor
150 kDa
170
130
mTOR
289 kDa
170
Chchd10
14 kDa
15
GAPDH
37 kDa
40
35
C
STC
HFD3d
TDP43
43 kDa
55
40
p62
62 kDa
55
Chchd10
14 kDa
15
HSP90
90 kDa
130
D
siCTR
siTDP43
WT
KO
WT
KO
TDP43
43 kDa
40
Raptor
150 kDa
170
130
p-p62 (S351)
62 kDa
70
55
p62
62 kDa
70
55
Chchd10
62 kDa
15
GAPDH
37 kDa
35
E
siCTR
siTDP43
WT
KO
WT
KO
Keap1
60-64 kDa
70
55
GAPDH
37 kDa
40
25
NRF2
68 kDa
70
55
NPM1
38 kDa
40
35
